# Restrictive Strategy vs Usual Care for Cholecystectomy in Patients With Abdominal Pain and Gallstones: 5-Year Follow-Up of the SECURE Randomized Clinical Trial

**DOI:** 10.1001/jamasurg.2024.3080

**Published:** 2024-08-21

**Authors:** Daan J. Comes, Sarah Z. Wennmacker, Carmen S. S. Latenstein, Jarmila van der Bilt, Otmar Buyne, Sandra C. Donkervoort, Joos Heisterkamp, Klaas in’t Hof, Jan Jansen, Vincent B. Nieuwenhuijs, Pascal Steenvoorde, Hein B. A. C. Stockmann, Djamila Boerma, Joost P. H. Drenth, Cornelis J. H. M. van Laarhoven, Marja A. Boermeester, Marcel G. W. Dijkgraaf, Philip R. de Reuver

**Affiliations:** 1 Department of Surgery, Radboud University Medical Centre, Nijmegen, the Netherlands; 2 Department of Surgery, Flevoziekenhuis, Almere, the Netherlands; 3 Department of Surgery, Maasziekenhuis Pantein, Boxmeer, the Netherlands; 4 Department of Surgery, Onze Lieve Vrouwe Gasthuis, Amsterdam, the Netherlands; 5 Department of Surgery, Elisabeth-Tweesteden Ziekenhuis, Tilburg, the Netherlands; 6 Department of Surgery, Admiraal de Ruyter Ziekenhuis, Goes, the Netherlands; 7 Department of Surgery, Isala, Zwolle, the Netherlands; 8 Department of Surgery, Medisch Spectrum Twente, Enschede, the Netherlands; 9 Department of Surgery, Spaarne Gasthuis, Hoofddorp, the Netherlands; 10 Department of Surgery, Antonius Ziekenhuis, Nieuwegein, the Netherlands; 11 Department of Gastroenterology and Hepatology, Amsterdam UMC – Location AMC, Amsterdam, the Netherlands; 12 Department of Surgery, Amsterdam UMC – Location AMC, Amsterdam, the Netherlands; 13 Department of Epidemiology and Data Science, Amsterdam UMC – Location AMC, Amsterdam, the Netherlands

## Abstract

**Importance:**

The 1-year results of the SECURE trial, a randomized trial comparing a restrictive strategy vs usual care for select patients with symptomatic cholelithiasis for cholecystectomy, resulted in a significantly lower operation rate after restrictive strategy. However, a restrictive strategy did not result in more pain-free patients at 1 year.

**Objective:**

To gauge pain level and determine the proportion of pain-free patients, operation rate, and biliary and surgical complications at the 5-year follow-up.

**Design, Setting, and Participants:**

This randomized clinical trial was a multicenter, parallel-arm, noninferiority, prospective study. Between February 2014 and April 2017, patients from 24 hospitals with symptomatic, uncomplicated cholelithiasis were included. Uncomplicated cholelithiasis was defined as gallstone disease without signs of complicated cholelithiasis, ie, biliary pancreatitis, cholangitis, common bile duct stones, or cholecystitis. Follow-up data for this analysis were collected by telephone from July 11, 2019, to September 23, 2023.

**Interventions:**

Patients were randomized (1:1) to receive usual care or a restrictive strategy with stepwise selection for cholecystectomy.

**Main Outcomes and Measures:**

The primary, noninferiority end point was proportion of patients who were pain free as evaluated by Izbicki pain score at the 5-year follow-up. A 5% noninferiority margin was chosen. The secondary end points included cholecystectomy rates, biliary and surgical complications, and patient satisfaction.

**Results:**

Among 1067 patients, the median (IQR) age was 49.0 years (38.0-59.0 years); 786 (73.7%) were female, and 281 (26.3%) were male. At the 5-year follow-up, 228 of 363 patients (62.8%) were pain free in the usual care group, compared with 216 of 353 patients (61.2%) in restrictive strategy group (difference, 1.6%; 1-sided 95% lower confidence limit, −7.6%; noninferiority *P* = .18). After cholecystectomy, 187 of 294 patients (63.6%) in the usual care group and 160 of 254 patients (63.0%) in the restrictive strategy group were pain free, respectively (*P* = .88). The restrictive care strategy was associated with 387 of 529 cholecystectomies (73.2%) compared with 437 of 536 in the usual care group (81.5%; 8.3% difference; *P* = .001). No differences between groups were observed in biliary and surgical complications or in patient satisfaction.

**Conclusions and Relevance:**

In the long-term, a restrictive strategy results in a significant but small reduction in operation rate compared with usual care and is not associated with increased biliary and surgical complications. However, regardless of the strategy, only two-third of patients were pain free. Further criteria for selecting patients with uncomplicated cholelithiasis for cholecystectomy and rethinking laparoscopic cholecystectomy as treatment is needed to improve patient-reported outcomes.

**Trial Registration:**

CCMO Identifier: NTR4022

## Key Points

**Question:**

What is the long-term impact of restrictive selection or usual care for cholecystectomy in patients with symptomatic cholelithiasis in terms of persistent pain and biliary and surgical complications?

**Findings:**

This randomized clinical trial showed a reduction in operation rate after a restrictive strategy compared with usual care for patients with abdominal pain and gallstones. A restrictive strategy was not associated with increased biliary or surgical complications, and patient-reported outcomes in pain and symptomatology were not significantly different between the arms.

**Meaning:**

In patients with abdominal pain and gallstones, a more restrictive approach could be adopted to avoid unnecessary cholecystectomies.

## Teaser

This randomized clinical trial examines follow-up data for patients with symptomatic cholelithiasis to gauge pain level and determine the proportion of pain-free patients, operation rate, and biliary and surgical complications between patients who received usual care vs a restrictive strategy.

## Introduction

Laparoscopic cholecystectomy is the predominant treatment for patients with abdominal pain and gallstones, resulting in approximately 700 000 operations in the United States each year.^1^ While complicated cholelithiasis (ie, cholecystitis, choledocholithiasis, biliary pancreatitis) is an indication for cholecystectomy, there is lack of consensus about patients with uncomplicated symptomatic cholelithiasis (cholecystolithiasis) for who will benefit from surgery.^2,3^ To investigate the appropriateness and the benefit from a stepwise selection for cholecystectomy in patients with uncomplicated cholelithiasis, the Scrutinizing (In)efficient Use of Cholecystectomy, A Randomized Trial Concerning Variation in Practice, (SECURE trial) was initiated.^4^

The SECURE trial compared usual care vs a restrictive strategy with stepwise selection for laparoscopic cholecystectomy based on the Rome III criteria for biliary colic. Between 2014 and 2017, the trial included 1067 patients and showed that the primary outcome of pain reduction was suboptimal for both usual care and the restrictive strategy. The restrictive strategy was associated with a reduction in cholecystectomies by 7.7% compared with usual care, but noninferiority regarding patients without persistent pain was not demonstrated: 321 of 536 patients (59.9%) following usual care vs 298 of 529 (56.3%) after a restrictive strategy (noninferiority *P* = .32). Two other randomized trials compared pain in patients after cholecystectomy vs conservative treatment.^5,6^ A Norwegian single-center study (n = 137) investigated prevalence of symptomatic events and showed that a watchful waiting strategy was shown to be a feasible option in 31% of patients during 14 years of follow-up. A recent multicenter study in the UK randomizing 434 patients to receive conservative management or surgery showed that 25% of the participants in the conservative arm and 67% in the surgical arm had received surgery at the 18-month follow-up. While there was no difference observed in 36-item Short Form score (SF-36)–based bodily pain score, results were constrained by the high number of patients who declined to participate and the short-term follow-up.^5^

The initial follow-up period of 1 year in the SECURE trial was pragmatic but may have been too short to yield the true outcomes in the long run. Patient crossover from conservative to surgical treatment and the occurrence of late biliary complications impair outcomes of a restrictive strategy. To ascertain the long-term consequences of a restrictive strategy in patients with gallstones, there is a need for long-term data. The primary objective of the present study was to evaluate the operation rate, pain, biliary and surgical complications, and patient satisfaction concerning treatment at the 5-year follow-up of the SECURE cohort.

## Methods

### Study Design and Participants

The design of the SECURE trial study has been reported previously (see the trial protocol and protocol amendments in Supplement 1, Supplement 2, and Supplement 3).^4,7,8^ In short, the SECURE trial was a multicenter, randomized, parallel-arm, noninferiority study performed in 24 academic and nonacademic centers in the Netherlands. The initial study and 5-year follow-up were approved by the medical ethics commission (METC: 2013-129) and boards of directors of all participating hospitals. All included patients provided written informed consent before participation in the trial. This trial was registered in the Netherlands National Trial Register (NTR4022). Research was performed in accordance with the ethical standards of the updated Helsinki Declaration of 2013. The Consolidated Standards of Reporting Trials (CONSORT) reporting guidelines were followed.

Eligible participants were patients aged 18 to 95 years with abdominal pain and ultrasound-proven gallstones who were referred to a surgical outpatient clinic to discuss cholecystectomy. The inclusion and exclusion criteria were previously reported in the trial protocol and initial study.

### Randomization and Masking

Patients were randomized (1:1) to receive usual care or restrictive strategy before their first visit at the surgical outpatient clinic. Randomization was stratified for center (academic vs nonacademic and high vs low volume), sex, and body mass index. At the first visit, the treating physician completed a digital triage instrument to evaluate whether the included patients fulfilled 5 prespecified selection criteria for symptomatic cholelithiasis: (1) severe pain attacks, (2) pain lasting 15 to 30 minutes or longer, (3) located in epigastrium or right upper quadrant, (4) pain radiating to the back, and (5) a positive pain response to simple analgesics. These predefined selection criteria were formulated based on biliary colic (defined by Rome III criteria) with addition of pain radiating to the back or pain response to simple analgesics.^9,10,11^

### Study Group Allocation

Group allocation was revealed to patients and physicians only after completion of the triage instrument. In the usual care arm, result of the triage instrument was blinded, and treatment advice was not given. Patients assigned to the usual care arm received the standard care given in the participating centers, and selection for cholecystectomy was left to the discretion of the surgeon in shared decision with the patient.

In the restrictive strategy arm, advice to perform a laparoscopic cholecystectomy was displayed by the triage instrument for patients who fulfilled all 5 prespecified criteria of the triage instrument. Patients in the restrictive strategy arm who did not meet the prespecified criteria were selected for conservative treatment and for further workup in search of an alternative diagnosis for the abdominal symptoms.

### Outcomes

The primary end point in the SECURE study was the number of patients who were pain free at the 1-year follow-up. Pain free was defined as an Izbicki pain score (IPS) of 10 or less with a visual analog scale (VAS) pain score of 4 or less. The IPS is a validated pain score initially designed for chronic pancreatitis. It consists of 4 questions regarding the frequency of pain, intensity of pain, use of analgesics, and disease-related inability to work.^12^

Predefined secondary end points were number of cholecystectomies, complications due to gallstones or surgery-related complications classified according to Clavien-Dindo classification, and patient-reported satisfaction with the treatment measured using a numeric rating scale ranging from 1 to 10, with higher values indicating greater satisfaction.^13^ Lastly, the study explored additional health care utilization resulting from persistent abdominal pain, including return visits to outpatient clinics, additional imaging procedures, endoscopies, and concurrent identification of functional gastrointestinal disorder. Functional gastrointestinal disorder is a clinical diagnosis assigned by the treating physician when there are no other physical causes.

For the 5-year follow-up, 1 additional outcome was examined. Achieving a pain-free status was redefined as a VAS pain score of 4 or less. This definition for pain free, without the IPS, is based on a recent consensus meeting that include patients and care professionals.^14^

### Follow-Up

Long-term follow-up data were systematically retrieved from 3 different data sources from all patients included for the intention-to-treat analysis. Patients were contacted by telephone 5 years after enrolment. A structured telephone interview was conducted about persisting abdominal pain (yes/no), intensity of pain (VAS pain score), relief of symptoms (yes/no), and treatment satisfaction (rating scale of 1-10). If the patient reported persisting abdominal pain, the 5 prespecified selection criteria were assessed.

Subsequently, an additional questionnaire was sent by email to all patients to evaluate details of the IPS, the 5 prespecified selection criteria, intensity of pain (VAS pain score), other abdominal symptoms (ie, nausea and vomiting, diarrhea, difficult defecation, acid burn, and bloated feeling), and patient-reported satisfaction.^12^

Clinical data on cholecystectomy and complications due to cholelithiasis or surgery in the period between the 1- and 5-year follow-ups were obtained from patients’ medical records. Data regarding findings on upper gastrointestinal endoscopy and diagnoses of functional gastrointestinal disorder related to persisting pain were collected. Medical records of patients referred to another participating hospital were scrutinized to ascertain health care utilization across both institutions. The Dutch reimbursement model integrates competitive private insurance for curative care with government regulation. Additionally, individuals are subject to a deductible excess policy, requiring them to cover a portion of their health care costs before they receive government assistance.

### Statistical Analysis

The power analysis, strategy for patient replacement, and details of the statistical analysis are previously described in the protocol and statistical analysis plan.^7,8^ For the present study, noninferiority of the restrictive strategy vs usual care was assessed with a 1-sided 95% confidence limit (CL) for the primary outcome of pain-free status, defined as an IPS or 10 or less in combination with a VAS pain score of 4 or less. Additionally, noninferiority was assessed with a 1-sided 95% CL for the outcome of pain-free status based on a VAS pain score of 4 or less. The primary analysis was done by a 1-tailed χ^2^ test, comparing the proportions of pain-free patients at the 1-year follow-up between the usual care and restrictive strategy groups. Noninferiority was defined as the lower limit of the 1-sided 95% CL for the proportion of patients being pain free at 1 year after the restrictive strategy falling within the absolute 5% margin below the proportion under usual care. Both the intention-to-treat and per-protocol analyses were needed to demonstrate noninferiority of the restrictive strategy. Per-protocol analyses are presented in the eMethods in Supplement 4.

Secondary end point analyses were done using χ^2^ tests for dichotomous data, an independent *t* test for normally distributed continuous data, and the Mann-Whitney *U* test for skewed continuous data. Testing for normality of data distributions was based on the Shapiro-Wilks test. To assess the secondary outcomes after 5 years between patients with and without cholecystectomy, an additional comparison between these groups was performed. For secondary outcomes, statistical superiority tests and s-sided 95% CIs were reported. A *P* value less than .05 was considered significant. Missing data from the primary and secondary outcome after 5 years were not imputed since the response rate was more than 80%. Data analysis was performed using SPSS version 27.0 (IBM).

## Results

### Patients

Between July 11, 2019, and September 23, 2023, all 1067 included patients who passed the 5-year follow up were contacted by telephone. Their median (IQR) age was 49.0 years (38.0-59.0 years); 786 (73.7%) were female, and 281 (26.3%) were male. In total, 970 patients completed the telephone survey: 491 of 537 patients (91.4%) in the usual care group and 479 of 530 patients (90.4%) in the restrictive strategy group. No significant differences were found in patient characteristics (ie, sex and age) regarding responders and nonresponders. The questionnaire sent by email was completed by 716 of 1067 patients (67.1%): 67.6% in usual care group (363/537) vs 66.6% in restrictive strategy group (353/530).

Informed consent for follow-up after 1 year was withdrawn by 8 patients, resulting in a clinical data collection from 1059 of 1067 patients (Figure). Patient and baseline characteristics were published previously and are shown in Table 1.

**Figure.**
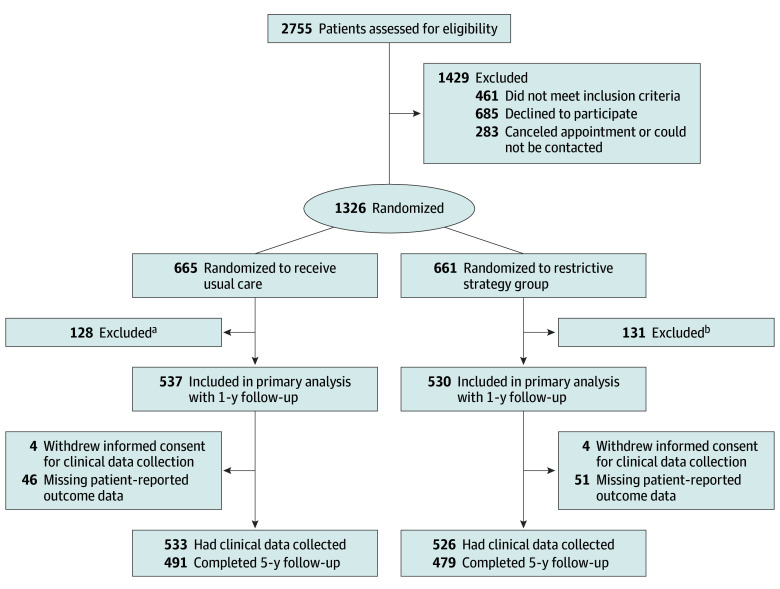
CONSORT Diagram Inclusion criteria included being referred to a surgical outpatient clinic, age 18 to 95 years, and having abdominal pain and ultrasonically confirmed gallstones. ^a^Patients were excluded from the usual care arm for the following reasons: 49 had missing data for baseline triage instrument, 36 withdrew informed consent, 20 were included in error, 7 had pregnancy or cancer at baseline, and 16 were lost to follow-up. ^b^Patients were excluded in from the restrictive strategy arm for the following reasons: 49 had missing data for baseline triage instrument, 38 withdrew informed consent, 29 were included in error, 5 had pregnancy or cancer at baseline, and 10 were lost to follow-up.

**Table 1.** Baseline Characteristics of the Intention-to-Treat Population

Charateristic	No. (%)		*P* value
	Usual care (n = 537)	Restrictive strategy (n = 530)	
Age, median (IQR), y	49.0 (39.0-58.0)	48.0 (37.0-59.0)	.56
Sex			.23
Female	387 (72.1)	399 (75.3)	
Male	150 (27.9)	131 (24.7)	
BMI, median (IQR)^a^	27.5 (24.6-31.2)	27.5 (24.5-30.9)	>.99
ASA class II	86 (16.0)	81 (15.3)	.74
History of abdominal surgery	205 (38.2)	196 (37.0)	.69
Use of pain medication	259 (48.2)	268 (50.6)	.58
Abdominal pain			
Izbicki pain score, median (IQR)^b^	35.5 (29.0-41.4)	35.0 (28.9-42.5)	.87
VAS pain score, median (IQR)^c^	7.5 (5.4-8.7)	7.5 (5.5-8.8)	.48
Biliary symptoms			
Severe pain in attacks^d^	411 (76.5)	440 (83.0)	.008
Located in right upper quadrant or epigastric region^d^	482 (89.8)	493 (93.0)	.06
Pain radiating to the back^d^	360 (67.0)	364 (68.7)	.57
Pain responding to simple analgesics^d^	289 (53.8)	284 (53.6)	.22
Duration of pain >15-30 min^d^	436 (81.2)	447 (84.3)	.17
Fulfilment of all 5 criteria^d^	152 (28.3)	201 (37.9)	.001
Functional symptoms			
Pain more than twice a month	357 (66.5)	328 (61.9)	.12
Intolerance of fat foods	243 (45.3)	250 (47.2)	.53
Nausea and vomiting	308 (57.4)	327 (61.7)	.15
Diarrhea	97 (18.1)	92 (17.4)	.76
Difficult defecation	102 (19.0)	97 (18.3)	.77
Acid burn	169 (31.5)	163 (30.8)	.80
Abdominal bloating	277 (51.6)	262 (49.4)	.48

Abbreviations: ASA, American Society of Anesthesiologists; BMI, body mass index; IPS, Izbicki pain score; VAS, visual analog scale.

^a^ Calculated as weight in kilograms divided by height in meters squared.

^b^ Imputed using multiple imputation with predictive mean matching; 103 missing values before imputation.

^c^ Imputed using multiple imputation with predictive mean matching; 101 missing values before imputation.

^d^ The 5 prespecified criteria for symptomatic cholecystolithiasis in the triage instrument.

### Long-Term Outcomes After Usual Care vs Restrictive Strategy

At the 5-year follow-up, 228 of 363 patients (62.8%; 95% CI, 57.8% to 67.8%) in the usual care group were pain free compared with 216 of 353 patients (61.2%; 95% CI, 56.1% to 66.3%) in the restrictive care group (difference, 1.6%; 1-sided 95% lower CL, −7.6%; noninferiority *P* = .18). The proportion of patients who were pain free after cholecystectomy was 187 of 294 patients (63.6%) in the usual care group and 160 of 254 patients (63.0%) in the restrictive care group (*P* = .88). A VAS pain score of 4 or less was reported in 397 of 491 patients in the usual care group (80.9%; 95% CI, 77.4% to 84.3%) vs 380 of 479 patients in the restrictive strategy group (79.3%; 95% CI, 75.7% to 82.9%; difference, 1.6%; 1-sided 95% lower CL, −5.7%; noninferiority *P* = .09). The results of the noninferiority test in the per-protocol analyses are detailed in the eResults in Supplement 4.

### Treatment and Safety Outcomes

The restrictive strategy resulted in 8.3% fewer cholecystectomies vs usual care (387/529 [73.2%] vs 437/536 [81.5%], respectively; *P* = .001). There were no significant differences in surgery- or cholelithiasis-related complications (Table 2). Patient-reported outcomes in terms of biliary and functional gastrointestinal symptoms were similar (Table 3). There was no significant difference observed in patient-reported satisfaction between both groups (median rating, 9.0 [IQR, 6.6-10] vs 8.9 [IQR 6.7-10.0]; *P* = .38). Lastly, because of persistent abdominal pain, additional health care was utilized in the overall SECURE population in terms of return visits to outpatient clinics (166/1059; 15.7%), extra imaging procedures (175/1059; 16.5%), endoscopies (49/1059; 4.6%), and the diagnosis functional gastrointestinal disorder (72/1059; 6.8%). The description of outcomes for patients with and without cholecystectomy is in the eResults and eTable in Supplement 4.

**Table 2.** Primary and Secondary Outcomes at the 1-Year and 5-Year Follow-Up of the Intention-to-Treat Population^a^

Outcome	At 1 y, No. (%)		*P* value	At 5 y, No. (%)		*P* value
	Usual care	Restrictive strategy		Usual care	Restrictive strategy	
Patient-reported						
Pain free^b^^,^^c^	321 (59.9)	298 (56.3)	.32^d^	228 (62.8)	216 (61.2)	.18^d^
VAS score ≤4	426 (79.3)	391 (73.8)	.59^d^	397 (80.9)	380 (79.3)	.09^d^
Cholecystectomy	404 (75.4)	358 (67.7)	.005	437 (81.5)	387 (73.2)	.001
Pain free^b^^,^^c^	256 (63.0)	228 (68.0)	.93	187 (63.6)	160 (63.0)	.88
VAS score ≤4^e^	337 (83.4)	289 (80.7)	.33	326 (80.9)	283 (80.4)	.86
Time to cholecystectomy, median (IQR), wk	6.0 (2.25-11.0)	6.0 (3.0-10.0)	.74	6.0 (3.0-13.0)	6.0 (3.0-13.0)	.78
Surgical complications	88 (21.8)	74 (20.7)	.77	100 (22.9)	81 (21.0)	.49
CDC <III	53 (60.2)	51 (68.9)		64 (64.0)	57 (70.4)	
CDC ≥III	35 (39.8)	23 (31.1)		36 (36.0)	24 (29.6)	
Gallstone complications	38 (7.1)	40 (7.6)	.16	77 (14.3)	79 (14.9)	.79
Choledocholithiasis	4 (0.7)	13 (2.5)		10 (1.9)	18 (3.4)	
Acute cholecystitis	10 (1.9)	5 (0.9)		16 (3.0)	12 (2.3)	
Biliary pancreatitis	4 (0.7)	3 (0.6)		5 (0.9)	6 (1.1)	
Cholangitis	0	0		0	0	
Colic with hospitalization^f^	20 (3.7)	19 (3.6)		46 (8.6)	43 (8.1)	
Gallstone complication preoperative	34 (6.3)	35 (6.7)	.81	59 (11.0)	53 (10.0)	.52
Patient-reported satisfaction, median (IQR)^g^	8.4 (8.0-9.0)	8.4 (8.0-9.1)	.98	9.0 (6.6-10.0)	8.9 (6.7-10.0)	.39

Abbreviations: CDC, Clavien-Dindo classification; IPS, Izbicki pain score; VAS, visual analog scale.

^a^ Data on the primary outcome (Izbicki pain score) were missing for 174/537 patients (32.4%) in the usual care group and 170/530 patients (32.1%) in the restrictive strategy group. Data on secondary outcomes were missing from patients’ medical records for 4/537 patients in the usual care group and 4/530 patients in the restrictive strategy group.

^b^ Pain free was defined as Izbicki pain score ≤10 and VAS pain score ≤4.

^c^ Assessed in 716 patients.

^d^ *P* value for noninferiority.

^e^ Assessed in 970 patients.

^f^ Hospitalization was defined as patient presentation at the emergency department and/or admission.

^g^ Satisfaction was reported by patients using a numeric rating scale ranging from 1 to 10, with higher values indicating greater satisfaction.

**Table 3.** Patient-Reported Outcomes and Health Care Consumption at 5-Year Follow-Up^a^

Outcome	At 5 y, No. (%)		*P* value
	Usual care	Restrictive strategy	
**Patient-reported outcomes**			
Biliary symptoms			
Severe pain in attacks^b^	90 (18.3)	80 (16.7)	.32
Located in the right upper quadrant and/or epigastrium	81 (16.5)	71 (14.9)	.24
Pain radiating to the back	46 (9.4)	50 (10.5)	.33
Pain responding to simple analgesics	22 (4.5)	17 (3.6)	.52
Duration of pain >15-30 min	75 (15.3)	79 (16.5)	.14
Fulfilment of all 5 criteria^c^^,^^d^	14 (2.9)	12 (2.5)	.78
Functional symptoms			
Intolerance of fat foods	49 (9.8)	46 (9.6)	.53
Nausea and vomiting	21 (4.3)	22 (4.6)	.85
Diarrhea	41 (8.4)	48 (10.0)	.52
Difficult defecation	34 (6.9)	24 (5.0)	.26
Acid burn	48 (9.8)	41 (8.6)	.43
Abdominal bloating	61 (12.4)	39 (8.1)	.01
Patient-reported satisfaction, median (IQR)^e^	9.0 (6.6-10.0)	8.9 (6.7-10.0)	.39
**Additional treatment and diagnosis**			
Visits to outpatient clinic with persistent abdominal symptoms^f^	84 (15.7)	82 (15.6)	.98
Surgery	41 (7.7)	48 (9.2)	.38
Gastroenterology	57 (10.7)	48 (9.2)	.42
Imaging (US and/or CT)	89 (16.7)	86 (16.4)	.91
Upper gastrointestinal endoscopy	25 (4.7)	24 (4.6)	.95
No findings	10 (40.0)	17 (70.8)	
GERD	9 (36.0)	5 (20.8)	
Barrett esophagus	1 (4.0)	1 (4.2)	
Esophagitis	3 (12.0)	0	
Esophagus carcinoma	0	1 (4.2)	
Diaphragmatic hernia	2 (8.0)	0	
Functional gastrointestinal disorders	39 (7.3)	33 (6.3)	.53

Abbreviations: CT, computed tomography; GERD, gastroesophageal reflux disease; US, ultrasound.

^a^ Data on secondary outcomes from patient-reported outcomes were available for 491 patients in the usual care group and 479 patients in the restrictive strategy group.

^b^ Assessed in 970 patients.

^c^ Assessed in 1059 patients.

^d^ The 5 prespecified criteria for symptomatic cholecystolithiasis in the triage instrument.

^e^ Satisfaction was reported by patients using a numeric rating scale ranging from 1 to 10, with higher values indicating greater satisfaction.

^f^ Total number of patients who visit either the Surgery outpatient clinic, Gastroenterology outpatient clinic, or both.

## Discussion

The 5-year follow-up from the SECURE trial illustrated that laparoscopic cholecystectomy offers a mediocre solution for patients experiencing symptomatic cholelithiasis in achieving a pain-free state. In the long term, a restrictive strategy resulted in a significant but small reduction in operation rate compared with usual care and was not associated with increased biliary and surgical complications. A restrictive strategy was equally disappointing as usual care for reaching a pain-free state after cholecystectomy. Overall, an 8.3% reduction in operation rate was observed after a restrictive strategy compared with usual care. A similar trade-off between cholecystectomies saved and pain-free patients was observed after 1 year. However, the patient-reported long-term treatment satisfaction between usual care and the restrictive strategy was not significantly different and increasingly converged over time. Therefore, the long-term outcome of this trial contends that the indications for gallbladder surgery in patients with uncomplicated cholelithiasis should still be critically examined.

Both short- and long-term results of the SECURE trial indicate that surgery represents a poor solution for certain patients presenting symptoms attributable to gallstones. Based on the used definition of pain-free state, persisting pain is reported in 20% to 40% of patients after cholecystectomy. The recently published C-GALL trial accords with our findings and supports the hypothesis that observation/conservative management is an alternative strategy to surgery.^5^ This study assessed whether cholecystectomy is cost-effective compared with observation/conservative management at 18 months. The score on the bodily pain domain of the SF-36 was the primary end point of the study. By 18 months, 25% in the observation/conservative management arm and 67% in the cholecystectomy arm had received surgery. The mean (SD) SF-36 norm-based bodily pain score was 49.4 (11.7) and 50.4 (11.6), respectively.^5^ The long-term outcome of the trial needs to show whether the lower operation rate is due to waiting lists in the UK and whether observation is sustainable in terms of biliary complications and patient-reported outcomes. Additionally, after given detailed information on the alternative options for cholecystectomy, many patients in the C-GALL opted for nonsurgical treatment and even declined participation in the trial to not receive surgery. This observation advocates shared decision making in selecting patients for cholecystectomy.

Since the initiation of the SECURE trial, more insights have been gained into the factors contributing to the limited efficacy of surgery. Last year, an international consensus was published on prioritizing outcomes relevant to all stakeholders, including clinicians and patients, and recently a decision rule has been validated to better assess the likelihood of achieving a pain-free state.^14,15^ Although cholecystectomy might provide relief from biliary colic, it does not treat nonbiliary abdominal pain and symptoms. Several studies showed the high prevalence of functional dyspepsia and/or irritable bowel syndrome in gallstone patients. More than a third of patients with gallstones eligible for cholecystectomy comply with the Rome IV criteria for these functional gastrointestinal disorders.^16,17^ The 6-month follow-up of 401 patients showed that patients with functional gastrointestinal disorders undergoing cholecystectomy were 2.5 times less likely to be pain free, compared with patients without these disorders. For patients with signs of functional disorders and gallstones, we advocate a follow-up period, for evaluating symptoms and reconsideration of surgery, for instance, after 4 weeks. During this period, patients should monitor their symptoms carefully. If functional gastrointestinal symptoms dominate their pain, these should be treated before cholecystectomy. The watchful waiting approach is relatively safe because the risk of developing complicated gallstone disease within this interval is lower than 2.5%.

The hampering impact of functional gastrointestinal disorders on the outcome of surgery accords with the variables included in a recently constructed decision tool to assess the likelihood of achieving a pain-free state. This tool was developed in 494 patients and validated in 1067 patients of the SECURE trial.^4,15^ After internal and external validation, the tool was able to discriminate well between patients with and without clinically relevant pain reduction when considering the following characteristics: age, history of abdominal surgery, VAS pain score, and the presence of nausea and/or heartburn. Lower pain scores and the presence of heartburn reduce the likelihood of achieving a pain-free state.

A pain-free state after surgery is one of the requirements to achieve textbook outcome (TO) after cholecystectomy according to an international consensus-based definition of TO. The definition was the result of expert meetings and surveys of patients and an international group of surgeons and gastroenterologists. The survey included responses from 490 Dutch patients and 603 clinicians representing 81 countries. The results of the surveys, combined with the findings from a conclusive consensus meeting, have established the TO as the absence of recurrent biliary colic leading to hospitalization, a reduction or absence of abdominal pain (VAS score ≤4), and the absence of biliary and surgical complications. A post hoc analysis of data from 1561 Dutch patients with uncomplicated gallstone disease revealed that 64% of patients achieved TO, primarily because a large percentage of the patients did not attain a pain-free state.^14^ Higher incidence of diarrhea or fatty food intolerance was observed in the group of patients who underwent cholecystectomy compared with those who did not. These are well-known consequences after cholecystectomy.^18^ The long-term metabolic impact of cholecystectomy on bile acid regulation and metabolic diseases is less well investigated.

In the design and initiation of the SECURE trial, our group extensively deliberated on the definition of pain free. We concluded that solely considering pain on a VAS score would not constitute a comprehensive outcome and that the use of analgesics and disease-related inability to work are also relevant outcome measures. Therefore, at that time, a definition of pain free was chosen, requiring patients to have an IPS of 10 or less in addition to a VAS pain score of 4 or less. The 5-year follow-up of the SECURE trial indicates that the inclusion of IPS has an adverse effect on the degree of pain-free outcomes.

One could argue that the IPS questionnaire is not validated for measuring persistent nonbiliary abdominal pain. If only a VAS pain score of 4 or less is considered, 80% of patients achieve pain-free status in both study arms. Despite discussions with patients during definition of the TO regarding analgesics and disease-related inability to work, these 2 outcome measures were not prioritized by patients or clinicians. This evolving insight has led to the decision to also report a VAS pain score of 4 or less as relevant outcome for patients with gallstones in the present 5-year follow-up.

### Limitations

There are some inherent limitations that need to be addressed for both the initial SECURE study and the current long-term follow-up. Approximately 30% of patients in the restrictive strategy did not adhere to the prescribed protocol. The majority of protocol deviations occurred in patients who underwent cholecystectomy without meeting the prespecified criteria of the triage instrument. The decision for cholecystectomy was often influenced by either the surgeon’s judgment or the patient’s preference, regardless of the triage instrument outcome. Furthermore, in the restrictive strategy, a higher proportion of patients with typical biliary symptoms, as per prespecified criteria, were included compared with the usual care arm. Consequently, the restrictive strategy cohort comprised a greater proportion of patients eligible for cholecystectomy than the usual care cohort. If this proportion had been equal in both arms, potentially even fewer cholecystectomies might have been performed in the restrictive strategy group compared with usual care.

A long-term follow-up results in more patients lost to follow-up. More than 90% of patients were contacted via telephone; however, only 70% completed the emailed survey, which included the IPS assessment. No correction for multiple comparisons was performed. Having 1 or 2 results indicating superiority may reflect false discoveries. Finally, the benefit of long-term testing for noninferiority when not demonstrated at 1 year could be questioned. We were interested in determining whether noninferiority was found given the decreasing deviation from the allowed 5% margin. Meanwhile, together with patients, the pain-free definition was redefined, and it appeared valuable to also assess this outcome for noninferiority. Based on the previous statement and regarding the intention-to-treat analyses, since a *P* value of .02 was observed solely in the per-protocol analyses, we cannot claim noninferiority of the restrictive strategy. Now it is left to the clinician to assess whether the initially set threshold of 5% is overly stringent.

Cost-effectiveness and the budget impact of a restrictive strategy were previously assessed and revealed that implementation of the restrictive strategy in subgroups of patients based on gender, body mass index, and hospital volume would result in national budgetary savings.^19,20^ Cost-effectiveness and the budget impact in the long term could motivate all stakeholders, including clinicians, health insurers, and government entities, to implement a restrictive strategy in specific groups of patients. Future studies should also include a lifestyle intervention as treatment for abdominal pain and gallstones to examine whether symptom relief could be achieved with fewer cholecystectomies. Such a study is currently under way in the Netherlands (NCT06002516) and could lead to a validated educational tool with lifestyle interventions for the best management for patients with upper abdominal pain, other gastrointestinal symptoms, and gallstones.

## Conclusions

Regardless of the strategy, only two-thirds of patients achieved a pain-free state after cholecystectomy. Although noninferiority could not be demonstrated over time, the restrictive strategy was not associated with an increase in biliary or surgical complications while resulting in fewer operations. The results of this long-term analysis may suggest that, in the future, a more restrictive approach could be adopted to avoid unnecessary cholecystectomies, and improving the selection of patients who actually benefit from cholecystectomy needs to be the focus of care.

## Notes

Supplement 1. Trial protocol

Supplement 2. Protocol amendment 1

Supplement 3. Protocol amendment 2

Supplement 4. **eMethods.** Non-inferiority testing in per protocol analysis **eResults.** Patients with and without cholecystectomy **eTable.** Comparison of patients with and without cholecystectomy after 5-year follow-up

Supplement 5. Data sharing statement
