# Supplementary material for: Restrictive Strategy vs Usual Care for Cholecystectomy in Patients With Abdominal Pain and Gallstones: 5-Year Follow-Up of the SECURE Randomized Clinical Trial
Source: JAMA Surg. 2024 Aug 21;159(11):1235–43. doi: 10.1001/jamasurg.2024.3080 (PMC11339699; doi:10.1001/jamasurg.2024.3080)
Supplement: Supplement 1. — Trial protocol [file jamasurg-e243080-s001.pdf]

The SECURE trial

Scrutinizing (in)efficient use of cholecystectomy: a randomized trial concerning variation in practice

PROTOCOL TITLE:

Scrutinizing (in)efficient use of cholecystectomy: a randomized trial concerning variation in practice

**Protocol ID**	**Not applicable**
**Short title**	**The SECURE trial**
**EudraCT number**	**Not applicable**
**Version**	**4**
**Date**	**Juli 2013**
**Coordinating investigator**	Dr. P.R. de Reuver, surgeon in training<br>Dept. Surgery<br>Academic Medical Center Amsterdam<br>P.O. Box 22660<br>1100 DD Amsterdam Zuid-Oost<br>p.r.dereuver@amc.uva.nl<br>Tel : 020-5669111
**Principal investigators**	Coordinating investigator, and in addition:<br><br>Prof. Dr. M.A. Boermeester, surgeon<br>Dept. Surgery<br>Academic Medical Center Amsterdam<br>P.O. Box 22660<br>1100 DD Amsterdam Zuid-Oost<br>m.a.boermeester@amc.uva.nl<br>Tel: 020-5669111<br><br>Prof. Dr. C.J.H.M. van Laarhoven, surgeon<br>Dept. Surgery<br>Radboud University Nijmegen Medical Center<br>P.O. Box 9101<br>6500 HB Nijmegen<br>k.vanlaarhoven@chir.umcn.nl<br>Tel: 024-3616421<br><br>Prof. Dr. J.P.H. Drenth, hepatologist<br>Dept. Gastroenterology and Hepatology<br>Radboud University Nijmegen Medical Center<br>P.O. Box 9101<br>6500 HB Nijmegen<br>j.drenth@mdl.umcn.nl<br>Tel: 024-3616999

	Prof. Dr. G.P. Westert, medical sociologist<br>Dept. IQ healthcare<br>Radboud University Nijmegen Medical Center<br>P.O. Box 9101<br>6500 HB Nijmegen<br>g.westert@iq.umcn.nl<br>Tel: 024-3619620<br><br>Prof. Dr. J.A. Roukema, surgeon<br>Dept. Surgery<br>St. Elisabeth Hospital Tilburg<br>P.O. Box 90151<br>5000 LC Tilburg<br>a.roukema@elisabeth.nl<br>mailto:k.vanlaarhoven@chir.umcn.nlTel: 013-5391313<br><br>Dr. B.L. den Oudsten, psychologist<br>Dept. Education and Research<br>St. Elisabeth Hospital Tilburg<br>P.O. Box 90151<br>5000 LC Tilburg<br>Dept. Medical and Clinical Psychology<br>Tilburg University<br>P.O. Box 90153<br>5000 LE Tilburg<br>b.l.denOudsten@uvt.nl<br>mailto:k.vanlaarhoven@chir.umcn.nlTel: 013-4662940<br><br>Dr. M.G. Dijkgraaf, methodologist<br>Clinical Research Unit<br>Academic Medical Center Amsterdam<br>P.O. Box 22660<br>1100 DD Amsterdam Zuid-Oost<br>m.g.dijkgraaf@amc.uva.nl<br>Tel: 020-5669111<br><br>Dr. D. Boerma, surgeon<br>Dept. Surgery<br>St. Antonius Hospital<br>P.O. Box 2500<br>3430 EM Nieuwegein<br>d.boerma@antoniusziekenhuis.nl<br>Tel: 088-3203000
**Grant support**	ZonMw<br>P.O. Box 93245

	2509 AE The Hague<br>Tel: 070-3495111
**Sponsors**	Academic Medical Center Amsterdam<br>Radboud University Nijmegen Medical Center<br>St. Elisabeth Hospital Tilburg<br>Tilburg University<br>St. Antonius Hospital Nieuwegein
**Subsidising party**	CZ healthcare insurer<br>P.O. Box 90152<br>5000 LD Tilburg<br>0900-0949
**Independent expert (s)**	Dr. R. Balm, vascular surgeon<br>Dept. Surgery<br>Academic Medical Center Amsterdam<br>P.O. Box 22660<br>1100 DD Amsterdam Zuid-Oost<br>r. balm@amc.uva.nl<br>Tel: 020-5669111
**Laboratory sites**	Not applicable
**Pharmacy**	Not applicable

PROTOCOL SIGNATURE SHEET

Name	Signature	Date
**Head of Department:**<br>Prof. Dr. D.A. Legemate., surgeon Dept. Surgery<br>Academic Medical Center Amsterdam<br>P.O. Box 22660<br>1100 DD Amsterdam Zuid-Oost		
**Coordinating Investigator**<br>Dr. P.R. de Reuver, surgeon in training Dept. Surgery<br>Academic Medical Center Amsterdam<br>P.O. Box 22660<br>1100 DD Amsterdam Zuid-Oost		
**Principal investigators**<br>Dr. P.R. de Reuver, surgeon in training Dept. Surgery<br>Academic Medical Center Amsterdam<br>P.O. Box 22660<br>1100 DD Amsterdam Zuid-Oost<br><br>Prof. Dr. M.A. Boermeester, surgeon Dept. Surgery<br>Academic Medical Center Amsterdam<br>P.O. Box 22660<br>1100 DD Amsterdam Zuid-Oost<br><br>Prof. Dr. C.J.H.M. van Laarhoven, surgeon Dept. Surgery<br>Radboud University Nijmegen Medical Center<br>P.O. Box 9101<br>6500 HB<br>Nijmegen<br><br>Prof. Dr. J.P.H. Drenth, hepatologist Dept. Gastroenterology and Hepatology Radboud University Nijmegen Medical Center<br>P.O. Box 9101<br>6500 HB<br>Nijmegen		

Dept. IQ healthcare<br>Radboud University Nijmegen<br>Medical Center<br>P.O. Box 9101<br>6500 HB<br>Nijmegen<br><br>Prof. Dr. J.A. Roukema,<br>surgeon Dept. Surgery<br>St. Elisabeth Hospital Tilburg<br>P.O. Box<br>90151 5000<br>LC Tilburg<br><br>Dr. B.L. den Oudsten,<br>psychologist Dept. Education<br>and Research<br>St. Elisabeth Hospital Tilburg<br>P.O. Box<br>90151 5000<br>LC Tilburg<br><br>Dr. M.G. Dijkgraaf,<br>methodologist Clinical<br>research unit<br>Academic Medical Center Amsterdam<br>P.O. Box 22660<br>1100 DD Amsterdam Zuid-Oost<br><br>Dr. D. Boerma,<br>surgeon Dept.		

TABLE OF CONTENTS

Inhoud

PROTOCOL TITLE: ... 2

PROTOCOL SIGNATURE SHEET ... 5

LIST OF ABBREVIATIONS AND RELEVANT DEFINITIONS ... 9

1. INTRODUCTION AND RATIONALE ... 12

2. OBJECTIVES ... 14

3. STUDY DESIGN ... 15

4. STUDY POPULATION ... 17

4.1 Population (base) ... 17

4.2 Inclusion criteria ... 17

4.3 Exclusion criteria ... 17

Definitions related to exclusion criterion 1: ... 18

Definitions related to exclusion criterion 5: ... 18

4.4 Sample size calculation ... 19

Feasibility ... 19

5. TREATMENT OF SUBJECTS ... 20

5.1 Investigational product/treatment ... 20

5.1 Use of co-intervention (if applicable) ... 20

5.2 Escape medication (if applicable) ... 21

6. INVESTIGATIONAL PRODUCT ... 22

6.1 Name and description of investigational product(s) ... 22

6.2 Summary of findings from non-clinical studies ... 22

6.3 Summary of findings from clinical studies ... 22

6.4 Summary of known and potential risks and benefits ... 22

6.5 Description and justification of route of administration and dosage ... 22

6.6 Dosages, dosage modifications and method of administration ... 22

6.7 Preparation and labelling of Investigational Medicinal Product ... 23

6.8 Drug accountability ... 23

7. NON-INVESTIGATIONAL PRODUCT ... 24

7.1 Name and description of non-investigational product(s) ... 24

7.2 Summary of findings from non-clinical studies ... 24

7.3 Summary of findings from clinical studies ... 24

7.4 Summary of known and potential risks and benefits ... 24

7.5 Description and justification of route of administration and dosage ... 24

7.6 Dosages, dosage modifications and method of administration ... 24

7.7 Preparation and labelling of Non Investigational Medicinal Product 24
7.8 Drug accountability 24
8. METHODS 25
8.1 Study parameters/endpoints 25
8.1.1 Main study endpoint 25
8.1.2 Secondary study endpoints 25
8.1.3 Other study parameters 25
8.2 Randomization, blinding and treatment allocation 26
8.3 Study procedures 26
8.4 Withdrawal of individual subjects 28
8.4.1 Specific criteria for withdrawal 28
8.5 Replacement of individual subjects after withdrawal 28
8.6 Follow-up of subjects withdrawn from treatment 28
8.7 Long term follow-up 28
8.8 Premature termination of the study 29
9. SAFETY REPORTING 30
9.1 Section 10 WMO event 30
9.2 AEs, SAEs and SUSARs 30
9.2.1 Adverse events (AEs) 30
9.2.2 Serious adverse events (SAEs) 30
9.2.3 Suspected unexpected serious adverse reactions (SUSARs) 31
9.3 Annual safety report 31
9.4 Follow-up of adverse events 31
9.5 Data Monitoring Committee 31
10. STATISTICAL ANALYSIS Descriptive statistics 33
Outcome variables 33
Analysis 33
Cost-effectiveness 34
Budget impact analysis (BIA) 35
10.1 Primary study parameter(s) 37
10.2 Secondary study parameter(s) 37
10.3 Other study parameters 38
10.4 Regulation statement 39
10.5 Recruitment and consent 39
10.6 Objection by minors or incapacitated subjects 39
10.7 Benefits and risks assessment, group relatedness 39
10.8 Compensation for injury 40

10.9 Incentives 40
11. ADMINISTRATIVE ASPECTS, MONITORING AND PUBLICATION 41
11.2 Monitoring and Quality Assurance 41
11.4 Annual progress report 41
11.5 End of study report 41
11.6 Public disclosure and publication policy 42
12. REFERENCES 42

LIST OF ABBREVIATIONS AND RELEVANT DEFINITIONS

**AE**	**Adverse Event**
**ALAT**	**Alanine Aminotransferase**
**AMC**	**Amsterdam Medical Center**
**AR**	**Adverse Reaction**
**ASA**	**American Society of Anesthesiology**
**ASAT**	**Aspartate Aminotransferase**
**BIA**	**Budget Impact Analysis**
**CA**	**Competent Authority**
**CCMO**	**Central Committee on Research Involving Human Subjects; in Dutch: Centrale Commissie Mensgebonden Onderzoek**
**CEA**	**Cost-Effectiveness Analysis**
**CRP**	**C-Reactive Protein**
**CRU**	**Clinical Research Unit**
**CUA**	**Cost-Utility Analysis**
**DMC**	**Data Monitoring Committee**
**ERCP**	**Endoscopic Retrograde Cholangiopancreatography**
**EU**	**European Union**
**EudraCT**	**European drug regulatory affairs Clinical Trials**
**GCP**	**Good Clinical Practice**
**GEE**	**Generalized Estimating Equations**
**IB**	**Investigator's Brochure**
**IC**	**Informed Consent**
**ICER**	**Incremental Cost-Effectiveness Ratio**
**METC**	**Medical research ethics committee (MREC); in Dutch: medisch ethische toetsing commissie (METC)**
**NVGE**	**Dutch Gastroenterological Society; in Dutch: Nederlandse Vereniging voor Gastroenterologie (NVGE)**
**NVvH**	**Dutch Surgical Society; in Dutch: Nederlandse Vereniging voor Heelkunde (NVvH)**
**QALY**	**Quality Adjusted Life Year**
**RUQ**	**Right Upper Quadrant**
**(S)AE**	**(Serious) Adverse Event**
**Sponsor**	**The sponsor is the party that commissions the organisation or performance of the research, for example a pharmaceutical**

company, academic hospital, scientific organisation or investigator. A party that provides funding for a study but does not commission it is not regarded as the sponsor, but referred to as a subsidising party.

SUSAR **Suspected Unexpected Serious Adverse Reaction**

VAS **Visual Analogue Scale**

WBC **White Blood Cell**

Wbp **Personal Data Protection Act (in Dutch: Wet Bescherming Persoonsgevens)**

WMO **Medical Research Involving Human Subjects Act (in Dutch: Wet Medisch-wetenschappelijk Onderzoek met Mensen**

SUMMARY

Rationale: Five to 22 percent of the adult Western population has gallstones. Among them, 13 to 22 percent becomes symptomatic during their lifetime. Cholecystectomy (i.e. gallbladder removal) is the preferred treatment option for symptomatic cholecystolithiasis (i.e. painful gallstones) today. In The Netherlands more than 20,000 cholecystectomies are performed annually against direct hospital-related costs of 55 million euro. Remarkably, gallbladder removal appears to be ineffective in 30-40% of patients. In addition, the Dutch health care insurance companies have noted a considerable practice variation in gallbladder removals attributable to a lack of evidence and to preferences that differ by surgeon.
Objective: In this prospective study we will examine the effectiveness of usual care with a restrictive care strategy using a standardized work-up with stepwise selection for cholecystectomy in patients with ultrasound proven gallstones and abdominal complaints over a 12 month period.
Study design: Randomized controlled trial.
Study population: Patients with abdominal pain and gallbladder stones or sludge
Intervention: Patients will be randomized (1:1) into two groups. Group 1 will be treated as usual. Treatment decisions will be based on the physician’s knowledge, preference, and experience. As a consequence, practice variation will be present in this group. Group 2 will be treated by using a strategy comprising interval evaluation with stepwise selection for laparoscopic cholecystectomy. In this stepwise selection, patients strictly meeting the criteria for symptomatic cholecystolithiasis according to the Dutch national guideline Gallstones will be offered a laparoscopic cholecystectomy. Patients not meeting the criteria will be re-evaluated at 3-month intervals.
Main study endpoints: Effectiveness is defined as the comparison of usual care with the restrictive strategy on the proportion of patients being pain free after 12 months
Nature and extent of the burden and risks associated with participation, benefit and group relatedness: The gallbladder will be removed in both groups if there is a clear medical reason. A burden to patients may be that they could prefer to be operated immediately; however, there is a possibility that we postpone the cholecystectomy or do not perform surgery if the symptoms have disappeared after a period of time. Patients will be asked to complete a booklet with questionnaires on symptoms and well-being. Patients will complete the questionnaires before treatment and at 3, 6, 9 and 12 months after inclusion. Questionnaire completion will take about 15 minutes per assessment.

1. INTRODUCTION AND RATIONALE

Five to 22 percent of the adult Western population has gallstones.[1, 2] The majority of the patients with gallstones remain asymptomatic. About 13% to 22% of the patients with gallstones will become symptomatic during their lifetime.[3, 4] Yearly, this corresponds to approximately 28,000 patients in The Netherlands, who are diagnosed with symptomatic cholecystolithiasis.[5] Symptomatic cholecystolithiasis is complicated by choledocholithiasis, acute cholecystitis, acute pancreatitis or cholangitis in less than 2% of cases annually.[6] Removal of both gallstones and the gallbladder (i.e. cholecystectomy) is the first choice of treatment by removing the organ that both contributes to formation of gallstones as well as the complications ensuing from them.[7]

In patients with uncomplicated symptomatic cholecystolithiasis a cholecystectomy is also often being performed. The original purpose of this operation in this group of patients is to relieve symptoms.[7] The symptoms associated with symptomatic gallstone disease consist of biliary colic defined as a severe steady pain, lasting 15-30 minutes or more, usually located in epigastrium and/or right upper quadrant, pain radiating to the back right and a positive reaction to simple analgetics. However, many patients scheduled for cholecystectomy have less specific and more debatable indications for surgery.[8-11]

The Dutch health care insurance companies have reported a considerable practice variation in cholecystectomies in The Netherlands.[12] Their report of the Dutch Health Care Insurers shows that the number of cholecystectomies varied from 39 to 609 cholecystectomies per centre per year. In addition, the variation in cholecystectomies performed per 100,000 insured inhabitants varied from 48 to 262 procedures. Because laparoscopic cholecystectomy is a common surgical procedure, which is performed in the regional hospitals covering its catchment area, a centralization effect cannot explain this variation. Moreover, the total number of cholecystectomies increased from 12,000 to 20,000 per year since 1990.[5] This increase in the number of performed procedures is consistent with the worldwide prevalence of cholecystectomy. The increase in cholecystectomies parallels the introduction of laparoscopy.[13] The indication for gallbladder removal is not based on evidence from randomized research and not restricted within guidelines. The current incentive to operate lies in patient's demands and the economic interest of the care provider.

In The Netherlands, about 23,000 cholecystectomies are performed each year.[5] However, a large part of the patients experience pain after the gallbladder removal.[14] There is some lower level of evidence suggesting that persistence of pain after cholecystectomy varies between 10% and 40%.[15-21] This would mean that approximately 2,300 up to 9,200 patients are ineffectively treated annually. Postoperative pain is the main predictor of a patient reported unsuccessful outcome after a cholecystectomy according to a large prospective cohort study.[21]

In addition, laparoscopic cholecystectomy is associated with 5.5% morbidity and 0.2% mortality. Bile duct injury is the most serious procedure-specific complication and occurs in 0.5 to 1% of procedures.[22, 23] Bile duct injury is a feared complication of a cholecystectomy and associated with increased morbidity and poor long-term quality of life. Several studies showed that this complication is a health and monetarily burden with severe long term morbidity in generally young patients and associated with high rates of litigation claims.[22, 23]

These ineffective operations and additional complications generate costs (both direct as well as indirect costs) for society. The direct – hospital related – costs of cholecystectomies are about 55 million euro a year.[24] More than 60% of the total costs of employed patients are caused by indirect costs related to sick-leave of employees.[25] In successful laparoscopic cholecystectomy time before return to work ranges from 1 up to 10 weeks.[26] Consequently, the total costs related to cholecystolithiasis are many times higher than 55 million euro.

A standardized work-up is lacking in the guidelines of the Dutch Surgical Society (Nederlandse Vereniging voor Heelkunde; NVvH) and Dutch Gastroenterology Society (Nederlandse Vereniging voor Gastroenterologie; NVGE), nor available in American and European guidelines. This research aims to optimize the outcome of patients with gallstones and abdominal complaints by optimizing the indication for cholecystectomy. In this randomized trial current usual care will be compared with a restrictive strategy with standardized work-up and stepwise selection for cholecystectomy. We will evaluate if this stepwise selection for cholecystectomy is non-inferior with respect to the proportion of patients being pain free after 12 months compared to usual care.

Results of the present study will be used to define standards for indication for cholecystectomy. As a consequence, the results affect health care policy concerning cholecystectomy for the next decades. Moreover, it is expected that the use of more stringent selection procedures will lead to less ineffective cholecystectomies. Thus, this study may contribute to current and future political and governmental initiatives to keep health care affordable in our Dutch and perhaps other societies in the European Union. As such, the subject of research fully complies with current priority setting in health care efficiency policy measure with continuous attention for stepped care and standard of care approaches.[27]

2. OBJECTIVES

Primary Objective: To examine the effectiveness of usual care with a restrictive care strategy using a standardized work-up with stepwise selection for cholecystectomy in patients with ultrasound proven gallstones and abdominal complaints over a 12 month period.
Effectiveness is defined as the comparison of usual care with the restrictive strategy on:
The proportion of patients being pain free at 12 months follow-up

Secondary Objective(s): To compare usual care with the restrictive strategy on:
(a) The proportion of patients being pain free after cholecystectomy
(b) The proportion of cholecystectomies
(c) The proportion of patients with complications due to gallstones
(d) Health status
(e) Time to pain-free
(f) The relation between the patients' symptoms and treatment and work performance
(g) Cost-effectiveness
(h) The proportion of patients with complications due to a cholecystectomy

Additional Objective(s): To assess:
(a) The relationship between specific symptoms or sets of symptoms and being pain-free after cholecystectomy.
(b) The practice variation indicator defined as the number of patients with cholecystectomy on hospital level per 100,000 inhabitants in the catchment area of the hospital, adjusted for relevant patient characteristics (i.e. age, sex, socio-economic status).

3. STUDY DESIGN

This study is designed as a multi-center, randomized, controlled, parallel-arm, non-inferiority study in subjects with abdominal symptoms and who have ultrasound proven gallstones or sludge. When a referral letter of a patient meeting the eligibility criteria, reaches the outpatient department of the hospital, study information and a baseline questionnaire will be send to the corresponding patient. Subsequently, patients signing informed consent will be enrolled by the 19 participating academic and general hospitals. Subjects will be randomized between usual care and a more restrictive strategy of standardized work-up with stepwise selection. Central coordination of this study will be performed by the AMC Amsterdam. For a schedule of visits and timing of assessments, we refer to the study diagram (See Addendum 1).

With respect to outcome (i.e. abdominal pain, complications due to gallstones), it is assumed that the restrictive strategy is not inferior compared with usual care. However, we expect a lower number of cholecystectomies that will be performed in the restrictive strategy arm. The chosen study design best fits the focus of research in which optimal patient selection (identifying the right patient for the right treatment) rather than treatment effectiveness (identifying the best treatment given the right patient) is studied; estimates of both, the proportion of patients in whom unnecessary cholecystectomies can be prevented as well as the resulting overall effectiveness at the group level, automatically follow from this design.

Two alternative designs were considered, but discarded for several reasons. First, a design with randomization of patients to laparoscopic cholecystectomy or no surgery would focus exclusively on the effect of surgery itself and would be in need of a well-defined target population as reflected in selective inclusion and exclusion criteria. Such design would ignore the core issue in this proposal that practice variation and overconsumption of care result from the presence of indeterminate means of patient selection and indication.

Second, a cluster randomized design with hospitals (rather than patients) randomized to usual care or to the restrictive standardized work-up would run the risk of selection bias and confounding. Selection bias in a cluster randomized trial might occur, if the local recruitment of patients would be influenced by prior knowledge of the treatment strategy offered by the hospital; confounding following an uneven distribution of hospitals with similar characteristics over both trial arms might easily occur given the number of participating hospitals. In addition, an adequate informed consent procedure for such cluster randomized trial would be troublesome to accomplish. The current study design of randomizing patients to usual care or the restrictive standardized work-up does not exhibit the weaknesses of these alternative designs.

However, a potential pitfall is the risk of contamination of usual care by the restrictive standardized work-up approach as it is conceivable that specialists transfer their experience with the work-up to usual care. It should be noted however that the equipoise principle still holds for usual care and the standardized work-up. It is very unwise and perhaps even unethical to adjust usual care as long as the clinical non-inferiority and health economic superiority of the restrictive standardized work-up approach has not been demonstrated yet. The equipoise principle should make surgeons in the participating hospitals indifferent regarding their preference for either approach during the study period. To further counter the risk of contamination as well as its potential impact on the study results when contamination happens to emerge to some degree, we will monitor each step during usual care in the clinical report forms. Additionally, a sample size is calculated conditional on the presence of contamination (see under 4.4 Sample size).

4. STUDY POPULATION

4.1 Population (base)

Subjects will be drawn from the outpatient department of surgery visiting one of the 19 participating hospitals. A total of 1038 patients will be enrolled.

4.2 Inclusion criteria

In order to be eligible to participate in this study, patients should meet all of the following criteria:

1. Having abdominal symptoms and having ultrasound proven gallstones or sludge (proven before or after referral)
2. Being referred to a surgeon for the treatment of suspected symptomatic gallstone disease
3. Aged 18 years or older
4. Providing informed consent

4.3 Exclusion criteria

Potential participants meeting any of the following criteria will be excluded from this study:

1. History of complicated cholelithiasis (i.e. choledocholithiasis, acute cholecystitis, biliary pancreatitis or cholangitis) since these types of patients are scheduled for elective cholecystectomy to prevent recurrence of complicated cholelithiasis rather than to prevent complaints of symptomatic cholecystolithiasis;
2. Indication for primary open cholecystectomy;
3. History of current malignancy;
4. Expected short life span of less than 12 months;
5. Suffering from severe or life-threatening systemic diseases (American Society of Anesthesiologists (ASA) class III and IV);
6. Known cirrhosis of the liver;
7. Current schizophrenia, memory deficiency, or any other disorder that predispose them to unreliable questionnaire responses;
8. Mentally incompetent;
9. Insufficient knowledge of the Dutch language;
10. Known pregnancy;
11. Residence in a federal correctional institution;
12. Participation in other (experimental) trials investigating pharmaceutical agents or strategies aimed at intervening with the natural disease course.

Definitions related to exclusion criterion 1:

- Choledocholithiasis: Defined as endoscopic retrograde cholangiopancreatography (ERCP) proven gallstones in the common bile duct.
- Acute cholecystitis: Defined according to the 2007 Tokyo classification, grade I to III.[28]

A. Local signs of inflammation: (1) Murphy's sign, (2) right upper quadrant (RUQ) mass/pain/tenderness.

B. Systemic signs of inflammation: (1) Fever, (2) elevated C-reactive protein (CRP), (3) elevated white blood cell (WBC) count.

C. Imaging findings characteristic of acute cholecystitis.

Definite diagnosis

(1) One item in A and one item in B are rated positive.

(2) C confirms the diagnosis when acute cholecystitis is suspected clinically.

- Biliary pancreatitis: abdominal pain with serum amylase and/or lipase levels elevated to at least 3 times the institutional upper limit of normal and one of the following scenarios. Definition according to a previous publication.[29]

  A. Gallstones and/or sludge diagnosed on imaging (transabdominal or endoscopic ultrasound or CT).

  B. In the absence of gallstones and/or sludge, a dilated common bile duct on ultrasound (>8mm in patients <=75 years or 10 mm in patients >75 years).

  C. *In absence of the previous 2:* the following laboratory abnormality: Alanine aminotransferase (ALAT) level >2 times higher than normal values with an ALAT > aspartate aminotransferase level (ASAT).

- Cholangitis: All of the following features. Definition according to a previous publication.[29]

  A. Serum total bilirubin level >40µmol/L (>2.3 mg/dL) and/or dilated common bile duct (>6 mm) on transabdominal or endoscopic ultrasound or CT

  B. Temperature >38.5°C

Definitions related to exclusion criterion 5:

ASA classification

I. A normal healthy patient

II. A patient with mild systemic disease (e.g. well controlled hypertension)
III. A patient with severe systemic disease (e.g. morbid obesity, renal failure)
IV. A patient with severe systemic disease that is a constant threat to life
V. A moribund patient who is not suspected to survive without the operation

4.4 Sample size calculation

For the power analysis we assumed that in the restrictive arm the percentage of pain-free patients will be at least equal to the usual care arm at the end of follow-up. For the calculation we assumed - based on literature - that after usual care a maximum of 80% of patients will be pain-free.[14] If the restrictive strategy results in less than 75% of patients pain-free, then this strategy is considered inferior. Probably, the percentage of pain-free patients will be slightly better than the percentage of the usual care arm and rise to 82% or above. However, if the usual care arm becomes, to some extent, contaminated by the standardized work-up strategy, then the 80% pain-free estimate for the usual care arm will tend towards the 82% for the restrictive standardized work-up arm. If so, the boundary of non-inferiority should be increased as well in order to maintain the non-inferiority of 5%. Hence, if contamination would result in 81% of patients pain-free in the usual care arm, then the lower boundary of non-inferiority equals 76%. Although we do not expect contamination to happen, it will be accounted for in this calculation of the sample size. Thus, with a one-sided Z test, 80% power and a significance level of 5% a total of 1,038 evaluable patients (519 in each arm) needs to be included, if the boundary of non-inferiority equals 76%. In absence of contamination and a lower boundary of non-inferiority of 75%, this total number of 1,038 evaluable patients will result in a power of about 89%.

Feasibility

The logistic requirements have been set out within the multicenter collaboration of the previously ZonMw grant supported RELAP (232 patients)[30], OPTIMA (1021 patients)[31], DIABOLO (recruitment finished, included 540 patients) and SURPASS (over 6000 patients)[32] trials. A total of 19 hospitals, spread over almost all Dutch provinces are committed to participate. The average number of laparoscopic cholecystectomies performed in the 19 participating hospitals is more than 3000 annual; 1038 evaluable patients are needed. Therefore we assume that the three year timeline is feasible: 4 months run-in-time, 26 months inclusion period and follow-up of 1038 evaluable patients and 6 months data analyses and reporting.

5. TREATMENT OF SUBJECTS

5.1 Investigational product/treatment

The restrictive strategy includes a standardized work-up with stepwise selection for surgery. In this restrictive arm patients are stepwise selected for surgery after a specified history using a triage instrument based on the Rome criteria for biliary colic[8, 9] and systematic review of the literature.[10, 11] According to the Rome criteria a biliary colic is defined as a severe steady pain, lasting 15-30 minutes or longer, usually located in the epigastrium and/or right upper quadrant.[8, 9] Biliary colic defined according to the Rome criteria has shown to be insufficiently accurate for the diagnosis of symptomatic cholecystolithiasis.[24]

Systematic reviews of the literature showed that three symptoms have a significant relationship with the diagnosis of symptomatic cholecystolithiasis: biliary colic (OR 2.6, 95% CI 2.4 to 2.9), pain radiating to the back right (OR 2.8, 95% CI 2.2 - 3.7) and positive response to simple analgesics (OR 2, 95% CI 1.6 to 2.5).[10, 11] Also, according to the Dutch guideline Gallstones these symptoms are associated with symptomatic cholecystolithiasis.[24] Therefore two additional symptoms were added in the criteria for cholecystectomy in the restrictive arm of the study: pain radiating to the back and pain reduction after analgesics. Patients who meet these three criteria: biliary colic (Rome definition), pain radiating to the back, and pain reduction after analgesics are selected for primary gallbladder removal. Patients who do not meet all these criteria go for further work-up of alternative diagnoses and have an interval evaluation at the outpatient clinic every three months. Results of this work-up, symptoms and effect of therapy aimed at another likely diagnosis (derived from work-up) are repeatedly evaluated every three months during one year of follow-up.

At present times, this is the best available way of standardizing the decision making in the restrictive arm.[33] Furthermore, it is in this respect important to remember that in daily practice (usual care) such explicit standardized decisional process for selection of gallbladder removal is not yet made, hence its practice variation. The triage instrument may have some imperfection, but current practice is far from perfect.[12, 14, 34, 35]

5.1 Use of co-intervention (if applicable)

Participants are not allowed to participate in other (experimental) trials investigating pharmaceutical agents or strategies aimed at intervening with the natural disease course. Diagnostics, therapeutics or necessary extra outpatient clinic visits aimed to diagnose or

treat other possible diseases causing the abdominal symptoms is left to the discretion of the treating physician in both study arms.

5.2 Escape medication (if applicable)

Diagnostics such as a gastroscopy or therapeutics such as analgesics or antacids aimed to diagnose or treat other possible diseases causing the abdominal symptoms is left to the discretion of the treating physician in both study arms.

6. INVESTIGATIONAL PRODUCT

6.1 Name and description of investigational product(s)

The restrictive strategy includes a standardized work-up and multistage selection for cholecystectomy. Standardization is done by strict administration of the symptoms associated with symptomatic cholecystolithiasis as reported in the Dutch national guideline Gallstones.[24] In addition, the multistage selection includes an interval evaluation after every 3 months.

6.2 Summary of findings from non-clinical studies

Not applicable since only clinical studies have been performed.

6.3 Summary of findings from clinical studies

The effectiveness of the restrictive study arm is based on limited data. Gallstones are often discovered incidentally during ultrasonography and remain asymptomatic in nearly 80% of cases.[3, 4] After diagnosis, the risk of developing pain or complications from gallstones is low; about 2% annually.[6] Our group previously estimated in the model-based approach where patients without colics were offered no cholecystectomy, a reduction of cholecystectomies of more than 50%, but with an increase in complications.[33] Here we assume that some of these patients need surgery and can be selected after standardized triage and interval evaluation. Therefore, a restrictive strategy is more appropriate to examine than a primary non-surgical strategy.

6.4 Summary of known and potential risks and benefits

Potential risks are that symptoms may persist after a cholecystectomy or that complications may occur due to the surgery or due to the stones. Holding the equipoise principle for both strategy arms risks are expected to be equal, but against a lower number of cholecystectomies in the restrictive study arm.

6.5 Description and justification of route of administration and dosage

Not applicable

6.6 Dosages, dosage modifications and method of administration

Not applicable

6.7 Preparation and labelling of Investigational Medicinal Product

Not applicable

6.8 Drug accountability

Not applicable

7. NON-INVESTIGATIONAL PRODUCT

Not applicable.

7.1 Name and description of non-investigational product(s)

Not applicable

7.2 Summary of findings from non-clinical studies

Not applicable

7.3 Summary of findings from clinical studies

Not applicable

7.4 Summary of known and potential risks and benefits

Not applicable

7.5 Description and justification of route of administration and dosage

Not applicable

7.6 Dosages, dosage modifications and method of administration

Not applicable

7.7 Preparation and labelling of Non Investigational Medicinal Product

Not applicable

7.8 Drug accountability

Not applicable

8. METHODS

8.1 Study parameters/endpoints

8.1.1 Main study endpoint

Effectiveness is defined as the comparison of usual care with the restrictive strategy on:

The proportion of patients being pain free at 12 months of follow-up. Pain free is defined as a Izbicki Pain Score ≤10 (with a Izbicki visual analogue scale ≤ 4) over the last two weeks before evaluation.

8.1.2 Secondary study endpoints

A comparison of usual care with the restrictive strategy on:

(a) The proportion of patients being pain-free after cholecystectomy

(b) The proportion of cholecystectomies

(c) The proportion of patients with complications (i.e. choledocholithiasis, acute cholecystitis, biliary pancreatitis or cholangitis) due to gallstones.

(d) Changes in health status and valuation over time. Health status will be measured using generic and disease specific health status questionnaires after informed consent and after 3, 6 and 12 months.

(e) Time to pain free

(f) The relation between the patients' symptoms and treatment and work performance as reported in the Health and Labour questionnaire

(g) Cost-effectiveness (see for a detailed description paragraph 10).

(h) The proportion of complications due to the cholecystectomy. Surgical complications are classified according to the Clavien Dindo classification.[36]

8.1.3 Other study parameters

The following data will also be assessed:

(a) The relationship between specific symptoms or sets of symptoms and being pain-free after cholecystectomy.

(b) The practice variation indicator defined as the number of patients with cholecystectomy on hospital level per 100,000 inhabitants in the catchment area of the hospital, adjusted for relevant patient characteristics (i.e. age, sex, socio-economic status).

The following data will be obtained and registered by interviewing the patient at the outpatient clinic:

- age at first presentation;
- sex;
- weight;
- length;
- co-morbidity;
- surgical history;

8.2 Randomization, blinding and treatment allocation

Randomization will be computer- and web-based using stratification to ensure a balanced distribution of known possible confounders in both treatment groups, and in blocks of variable size. Randomization will be stratified according to the following characteristics: center, sex, and weight. To ensure allocation concealment the randomization list will be generated using an online computer software program (ALEA NKI-AVL, Amsterdam, The Netherlands, Release: 2.2.) and implemented into the web-based application.

8.3 Study procedures

For each subject, the duration of the clinical trial will be 12 months, including baseline visit, four 3-month interval visits up to 12 months of follow-up. A schedule of assessments is summarized in addendum 1.

Anonymous study number

Each patient will receive an anonymous study number.

Data collection at outpatient clinic

After informed consent and randomisation, subjects will visit the outpatient clinic. The following data will be collected from patient records: Age, sex, weight, length, co-morbidity, and surgical history. The completed baseline questionnaires will be collected from the patients. A patient randomized to the usual care arm will be selected for cholecystectomy based on the surgeon’s personal experience and preferences (Group 1). Patients randomized to the restrictive care arm (Group 2) will be selected for cholecystectomy if the patient’s complaints fulfil all symptoms that are associated with symptomatic cholecystolithiasis according to the Dutch national guideline Gallstones. If not all symptoms associated with symptomatic cholecystolithiasis are fulfilled, a subject will go for further work-up and will be re-evaluated after three months. This re-evaluation will be done at 3-month intervals up to 12 months of follow-up. Diagnostics, therapeutics or extra outpatient clinic visits aimed to diagnose or treat other possible diseases causing

the abdominal symptoms is left to the discretion of the treating physician in both study arms.

Questionnaires

The questionnaire booklet consist of the following parts (see Appendix II) and will be completed at baseline and at 3, 6, 9, and 12 months of follow-up:

- EuroQol 5 Dimensions (EQ-5D) and the Short-Form Health and Labour Questionnaire (SF-HLQ)[41]:The EQ-5D is a generic questionnaire, consisting of a Visual Analogue Scale (EQ-5D VAS) and a classification system (EQ-5D Profile).[37] The EQ-5D Profile covers five domains of health (mobility, self-care, usual activities, pain/discomfort and anxiety/depression), each with three levels of functioning: level 1, no problems; level 2, some problems; level 3, severe problems. The EQ-5D VAS is a graded, vertical line, anchored at 0 (worst imaginable health state) and 100 (best imaginable health state). The patient is asked to mark a point on the EQ-5D VAS that best reflects his/her actual health state. The SF-HLQ contains three modules covering absence from paid employment, production loss without absence from paid employment and impediments to paid of unpaid employment
- Gastrointestinal Quality of Life Index (GIQLI): This questionnaire includes both specific questions on gastrointestinal symptoms, for both the upper and lower digestive tracts, as well as generic questions on physical, emotional, and social capabilities.[38, 39] The GIQLI includes 5 domains: symptoms (19 questions), physical dysfunction (7 questions), emotional dysfunction (5 questions), social dysfunction (4 questions), and the effects of the medical treatment carried out (1 question). The 36 questions are answered using a response scale from 0 (worst appraisal) to 4 (best appraisal) for each question. Sum scores for each scale can be calculated as well as an overall score (ranging from 0 to 144 points): The higher the score, the higher the health status.
- The Izbicki Pain Score (IPS)[40]: This questionnaire is designed for upper abdominal pain and based on four questions regarding frequency of pain, intensity of pain (as indicated by a visual analogue score), use of analgetics and disease-related inability to work. This score ranges from 0-100, with higher scores indicating more severe pain.
- The Gallstone Symptom List[46]: This painscore was designed and used by a studygroup in 2012. This score was designed to asses symptoms belonging to symptomatic cholecystolithiasis.

8.4 Withdrawal of individual subjects

Patients can leave the study at any time for any reason if they wish to do so without any consequences. The investigator can decide to withdraw a subject from the study for urgent medical reasons. All data generated up to the time of discontinuation from the study will be analyzed and the reason(s) for discontinuation will be recorded if given.

8.4.1 Specific criteria for withdrawal

All patients have the right to withdraw at any point during treatment without prejudice. The investigator can discontinue a subject's participation in the trial at any time if medically necessary. In addition, subjects meeting the following criteria must be withdrawn from the trial:

- occurrence of any undercurrent illness which, in the opinion of the investigator, warrants the patient's permanent withdrawal from the trial;
- patients' noncompliance, defined as refusal of inability to adhere to the trial schedule
- at the request of the subject, investigator, or regulatory authority
- patient becomes pregnant
- patient is lost to follow-up

8.5 Replacement of individual subjects after withdrawal

In section 4.4 the sample size calculation for this study is given. Therefore an inclusion of 519 subjects per study group is aimed to generate a total of 1038 evaluable patients. If a subject discontinues from the study prematurely the reason if given must be fully evaluated and recorded appropriately in source documentation and the CRF.

8.6 Follow-up of subjects withdrawn from treatment

Patients withdrawn from treatment will always crossover into usual care. We will ask these patients if they will continue completing the questionnaires and if their medical record may be checked after 12 months of follow-up to verify if and what treatment they have received for their abdominal symptoms. Patients will be asked oral and written informed consent.

8.7 Long term follow-up

Five years after inclusion, patients will be approached to fill-out a long term follow-up questionnaire. Only the patients who gave permission on the informed consent to contact them again (after initial 1 year follow-up of the trial), will be send this questionnaire. The long term questionnaire will consist of the same questionnaires as 12 month follow-

up questionnaire (Izbicki Pain Score, The Gallstone Symptom List, EuroQol 5 Dimensions (EQ-5D), the Short-Form Health and Labour Questionnaire (SF-HLQ), Gastrointestinal Quality of Life Index (GIQLI)) and additional questionnaires on healthcare consumption for gallstones or persistent abdominal pain after cholecystectomy (These questionnaires are:

- Patients' Experience of Surgery Questionnaire (PESQ). The PESQ consists of general questions on results of surgery, abdominal symptoms and related healthcare use.
- Medical Consumption Questionnaire (iMCQ). The iMCQ is a non disease-specific questionnaire to assess (costs of) healthcare consumption, and can be specified to the relevance of a specific disease. In this study the iMCQ contains questions on healthcare consumption related to gallstone symptoms or persistent abdominal pain after LC both in primary care, secondary care and additional diagnostics, treatments and medication.
- Productivity Cost Questionnaire (iPCQ). The iPCQ assesses sick leave and productivity loss for paid and unpaid work. The questionnaire was specified for gallstone symptoms and persistent abdominal pain after LC.

With the 5 years follow-up, the effect of a restrictive strategy for gallbladder removal can be evaluated on long term. The primary and secondary endpoints as described for the 12 month follow-up of the trial, will be established at 5 year follow-up. Additional secondary outcomes are the healthcare consumption for gallstone symptoms and persistent abdominal symptoms after cholecystectomy.

~~8.7~~8.8 Premature termination of the study

Safety assessments will consist of monitoring and recording all complications due to surgery or due to the gallstones. An independent data monitoring committee (DMC) will be formed to review these safety data. This board will be managed by an independent statistical data analysis center (Clinical Research Unit (CRU), AMC Amsterdam). This board will advise the investigators to continue the study, to implement protocol changes or to terminate this study. The DMC will be guided by a charter defining their role and responsibilities.

9. SAFETY REPORTING

9.1 Section 10 WMO event

In accordance to section 10, subsection 1, of the WMO, the investigator will inform the patients and the reviewing accredited METC if anything occurs, on the basis of which it appears that the disadvantages of participation may be significantly greater than was foreseen in the research proposal. The study will be suspended pending further review by the accredited METC, except insofar as suspension would jeopardise the patients' health. The investigator will take care that all patients are kept informed.

9.2 AEs, SAEs and SUSARs

9.2.1 Adverse events (AEs)

See 9.2.2.

9.2.2 Serious adverse events (SAEs)

The coordinating investigator will be responsible to report (severe) adverse events and will act as necessary to report (serious) adverse events.

Adverse events are defined as any undesirable experience occurring to a patient during the study, whether or not considered related to the investigational product. All adverse events reported spontaneously by the patient or observed by the investigator or his staff will be recorded.

A serious adverse event (SAE) is any untoward medical occurrence or effect that:
- results in death;
- is life threatening (at the time of the event);
- requires hospitalisation or prolongation of existing inpatients' hospitalisation
- results in persistent or significant disability or incapacity

All SAEs will be reported through the web portal ToetsingOnline to the accredited METC that approved the protocol, within 15 days after the DMC has first knowledge of the serious adverse reactions. SAEs that results in death or are life threatening should be reported expedited. The expedited reporting will occur not later than 7 days after the coordinating investigator has first knowledge of the adverse reaction. This is for a preliminary report with another 8 days for completion.
A predefined list of SAEs (inclusive of fatal cases) will be reported, namely the following:

- Major morbidity needing conservative treatment and/or (re)admission:

a) The development of complicated cholelithiasis (i.e. choledocholithiasis, acute cholecystitis, biliary pancreatitis or cholangitis)

b) Renal failure

c) Myocardial infarction

d) Cerebrovascular incident

e) Respiratory failure

f) Ischemia or necrosis of an organ

g) Sepsis of any type

h) Death

- Major morbidity needing surgical intervention (including drainage):

a)The development of complicated cholelithiasis (i.e. choledocholithiasis, acute cholecystitis, biliary pancreatitis or cholangitis)

b) Bile duct inury

c) Intra-abdominal hematoma

d) Intra-abdominal bleeding (ie after surgery)

e) Intra-abdominal abcess,

f) Perforation of visceral organ (ie during surgery)

g) Wounddehiscence

The remaining SAEs are recorded in an overview list that will be submitted periodically (once a year) to the METC.

9.2.3 Suspected unexpected serious adverse reactions (SUSARs)

Not applicable.

9.3 Annual safety report

Not applicable

9.4 Follow-up of adverse events

Not applicable

9.5 Data Monitoring Committee

An independent DMC will be formed. This committee will be guided by a charter defining their role and responsibilities, and methods specific to the committee. The DMC is

managed by representatives of the statistical data analysis centre (CRU, AMC Amsterdam). The DMC will assess safety by analysis of the complication ratio due to surgery or due to the gallstones in both study arms (see DMC charter, safety analysis). The DMC will advise the study group to continue, to adapt or to terminate the study. The study group will oversee the design, conduct and analysis of the study. The advices of the DMC will be notified upon receipt by the Coordinating Investigator to the METC that approved the protocol. With this notification a statement will be included indicating whether the advice will be followed and if not, a rationale will be provided.

10. STATISTICAL ANALYSIS

Descriptive statistics

Demographic and baseline characteristics

Data on demographic and baseline characteristics will be summarized for continuous variables, in case of normal distribution by means and standard deviations, and in case of non-normal distribution by medians and interquartile ranges. For discrete variables data (eg, sex, socio-economic status) will be summarized by frequencies and proportions.

Outcome variables

The primary outcome variable is the number of patients being pain-free. Pain free is defined as a Izbicki Pain Score ≤10 (with a Izbicki visual analogue scale ≤ 4) over the last two weeks before evaluation.
The secondary outcome variables are the number of patients being pain free after cholecystectomy; the number of cholecystectomies after 12 months; complications related to cholecystolithiasis; health status scores; time to pain-free; sick leave and working disability as assessed by the HLQ; cost-effectiveness; complications due to the cholecystectomy. Additional variables are symptoms or sets of symptoms as assessed by the GIQLI and the Gallstone Symptom Score; the number of patients with cholecystectomy on hospital level per 100,000 inhabitants in the catchment area of the hospital. Results will be summarized for continuous variables, in case of normal distribution by mean and standard deviation, and in case of non-normal distribution by median and interquartile range. For discrete variables data (eg, percentage of patients pain-free) will be summarized by frequencies and proportions.

Analysis

The outcome measures as defined in this chapter (Chapter 10) will be analyzed. Considering the trial being a non-inferiority one, analyses will be carried out according to the intention-to-treat principle as well as the per protocol principle. For continuous data, student's t-test will be used to calculate differences between groups for normally distributed data or Mann-Whitney U test for non-normally distributed data. The χ^2 test will be used to compare dichotomized outcomes between the groups.
The generalized estimating equations (GEE) is a method that can be used for the analysis of repeated measurements. This procedure extends standard regression analysis, taking into account the correlation between measurements. GEE will be applied to study the impact of (i) centre, sex and weight on the probability of being pain-free at 12 months post-randomisation, and (ii) treatment strategy, centre, sex and weight on the number of cholecystectomies. To assess the relation between specific symptoms or sets

of symptoms and being pain-free at 12 months post-randomisation, logistic regression analyses will be performed. Data on quality of life will be assessed by repeated measurement analysis using a linear mixed model. In all analyses, statistical uncertainties are expressed in 95% two-sided confidence intervals. A p-value of <0.05 will indicate statistical significance.

Cost-effectiveness

General considerations

The economic evaluation will be undertaken as a cost-effectiveness analysis (CEA) with the costs per patient pain-free at 12 months as primary outcome measure. Additionally, a cost-utility analysis (CUA) will be performed with the costs per quality adjusted life-year (QALY) as outcome. The CEA closely relates to the results concerning the clinical outcome measure, the CUA is performed to enable priority setting during health care policy making across patient groups, interventions and health care settings. Both analyses will be performed from a societal perspective and the time horizon is set at 12 months. With this time horizon no discounting of costs and effects will be performed. Although the proportions of patients pain-free at 12 months is assessed under a non-inferiority hypothesis, we expect to observe a small trend in favor of the restrictive standardized work-up, which justifies the performance of cost-effectiveness analyses rather than cost-minimization analyses here.[42] Incremental cost effectiveness ratios (ICER) will be calculated in terms of extra costs per extra pain-free patient and extra costs per QALY gained. Results will be displayed graphically by means of cost-effectiveness planes and acceptability curves. Sensitivity analyses will be undertaken for plausible ranges in unit costs of cholecystectomy, for probability distributions of patients over different disease trajectories during follow-up, and for different national and international utility weights given the observed health states of patients. Subgroup analyses will be tentatively performed for hospitals with high and low pre-baseline volumes of cholecystectomies per 100,000 inhabitants of the adherence area covered. Also, subgroup analyses will be performed for difference by sex and weight categories.

Cost analysis

Direct and indirect medical and non-medical costs will be included while assessing the societal costs of the restrictive standardized work-up against usual care for patients with abdominal complaints and ultrasound proven gallstones or sludge. The direct and indirect medical costs include all costs of inpatient and outpatient hospital stay, major diagnostic and therapeutic procedures (particularly in the restrictive standardized work-up

approach), and consultations, to be collected digitally from hospital information systems and with clinical report forms. The costs of out-of-hospital care by general practitioner and paramedics as well as the direct non-medical out-of-pocket expenses (over-the-counter medication, informal care) and indirect non-medical costs of production loss from sick leave will be based on volume data gathered with repeat patient questionnaires at 3, 6 and 12 months post randomization. Unit costing will be based on the existing national guideline for costing in health care research. Additionally, the unit costs of cholecystectomy will be assessed for different levels of economies of scale. Unit costing of production loss will comply with the preferred valuation in The Netherlands, the friction cost method. Unit costs derived from different calendar years will be price-indexed for base year 2013 and expressed in Euros.

Patient outcome analysis

Whereas the presence or absence of cholecystolithiasis may heavily relate to a person's health we will use the GIQLI to assess changes in disease-specific health status. In addition, the EQ-5D questionnaire will be used at baseline and after 3, 6, and 12 months of follow-up to generate health status scoring profiles over time, which will be transposed into health utilities using population based tariffs of time trade-off ratings of health states. Based on the health utility scores over time, QALYs will be calculated by taking the product sum of the health utility scores and the periods in-between successive measurements during the 12 months of follow-up.

The budget impact analysis (Mauskopf guideline)[43], will generalize the measured volume reduction of cholecystectomies and production losses in the experimental arm to a national scale, based on annual incidence rates of patients with symptomatic cholecystolithiasis.

Budget impact analysis (BIA)

General considerations

The short- and mid-term affordability of the restrictive standardized work-up in patients with abdominal complaints in the presence of proven gallstones will be assessed from governmental, provider and insurer perspectives following a budget impact analysis.[43] Such analyses may guide reimbursement decisions on indications for cholecystectomy and may influence volume and price negotiations between insurer and health care provider. In this study, the budget impact analyses will be patient- rather than episode-based considering the indeterminate disease course in the year following first presentation among patients in whom cholecystectomy has been foregone. The analyses

will also be incidence-based including only new patients with abdominal complaints and gallstones. Consequently, linking the first year costs to yearly incidence data suffices to explore the impact on budgets.

The governmental perspective is chosen to help setting priorities in health care optimalization while simultaneously considering the wider implications of the restrictive standardized work-up beyond the health care sector (e.g. reduction of production losses). The governmental perspective further includes an assessment of budget impact across different categories of premium financed health care (e.g. institutions for specialized medical care, self-employed medical specialists, other curative health care). The current study proposal fits in well with projected national policy measures concerning health care efficiency by promoting stepped care and professional guidelines. The current study also supports policy measures to reduce unnecessary care and the associated costs (delineating reimbursement packages)[44]. The provider perspective is chosen to support local decisions on reallocation of surgeon time and operation room capacity. The insurer perspective is chosen to assess the net financial consequences of the introduction of restrictive standardized work-up and prevention of cholecystectomies. Whereas the numbers of cholecystectomies performed per 100,000 Dutch inhabitants vary considerably by geographical region, we expect that the prevention of unnecessary cholecystectomies may differentially affect the budgets of insurers, depending on their market share by region. The results of the current study may support insurers with relatively high cholecystectomy reimbursement volumes in setting ceiling volumes of cholecystectomy and/ or applying regressive reimbursement strategies in case of overproduction.[45] If proven cost-effective, the restricted standardized work-up needs further diffusion among health care providers. We expect that the reluctance to adopt the new work-up may be substantial in regions with relatively high cholecystectomy volumes because of the current pay-per-cholecystectomy incentive. As stated by our study co-financer CZ, the diffusion of the new work-up should probably be accompanied by a digital triage instrument to be applied by health care providers to each patient of the target population in order to get cholecystectomies reimbursed. Hence, the budget impact analysis will include the costs of implementing and maintenance of such triage instrument. The base case scenario of no change in usual care for patients with abdominal pain and proven gallstones will be assessed against alternative scenarios applying the restrictive standardized work-up according to the BIA flowchart (Figure 4). In the upper part the BIA flow chart shows the current mix of patient selection algorithms, in the lower part the restrictive work-up approach is presented. Each alternative scenario may differ from the base case scenario by the proportion of patients for whom immediate cholecystectomy may seem opportune and by the proportion of patients eventually in

need of cholecystectomy at a later stage. Six alternative scenarios, all including the suggested implementation costs, will be assessed: immediate, gradual, and partly adoption of the restrictive standardized work-up across the country, combined with whether or not hospitals' ranking by cholecystectomy volumes is taken into account.

Sensitivity analyses will be performed for restrictive pricing and ceiling volumes strategies as well as for the unit costs of cholecystectomy at different levels of economies of scale.

The budget impact analysis will be performed in MS Excel to allow for further analysis by health policy makers. The ultimate decision tool will include population, (regional) health care use, and cost modules, differentiated by patient selection algorithm (usual against restrictive standardized). The time horizon for all budget impact assessments will be 4 years and reported for each successive calendar year.

Cost analysis

For the budget impact analysis to be used for priority setting in health care, current unit costing guidelines for costing in health care research will be applied. In case of impact assessments concerning premium financed health care and from the health insurer perspective, existing tariffs will be used. For the provider perspective, both (local) real unit costs as well as existing tariffs will be used. A provisional budget impact assessment from a governmental perspective has been reported in the cost effectiveness section of this proposal. In short, the total savings of the first four years may range between 32.5 and 65 million euro, excluding the savings resulting from reduced sick-leave.

10.1 Primary study parameter(s)

The primary outcome variable is the proportion of patients pain-free at 12 months of follow-up. Pain free is defined as a Izbicki Pain Score ≤10 (with a Izbicki visual analogue scale ≤ 4) over the last two weeks before evaluation.

10.2 Secondary study parameter(s)

The secondary study parameters are:

- The proportion of patients being pain free after cholecystectomy. Pain free is defined as a Izbicki Pain Score ≤10 (with a Izbicki visual analogue scale ≤ 4) over the last two weeks before evaluation.
- The number of cholecystectomies after 12 months.

- The proportion (in percentages) of patients with complications related to cholecystolithiasis. These complications are defined as acute cholecystitis, cholangitis, choledocholithiasis and biliary pancreatitis. Definitions are reported in chapter 4.
- Changes in health status and valuation over time. Health status will be measured using the EQ-5D and GIQLI questionnaires.
- Time to pain-free in months using the Izbicki Pain Score.
- Working disability over time will be measured using the HLQ.
- Cost-effectiveness defined as costs (in Euros) per patient pain-free at 12 months. Additionally, CUA will be performed defined as the costs (in Euros) per QALY.
- Complications due to the cholecystectomy. Surgical complications are classified according to the Clavien Dindo classification.[36]

10.3 Other study parameters

- Abdominal symptoms and pain characteristics will be measured using the GIQLI and Gallstone Symptom List.
- age at first presentation in years
- sex (male or female)
- weight in kilograms;
- length in meters;
- co-morbidity for which the patient uses medication;
- abdominal surgical history;

ETHICAL CONSIDERATIONS

10.4 Regulation statement

The trial will be conducted according to the International Conference of Harmonization. Good Clinical Practice Guidelines and all other applicable regulatory requirements and adheres to the ethical principles that have their origin in the declaration of Helsinki. Patient privacy is ensured by de-identifying all submitted data and using a subject identification code. All patients will have the right to withdraw from the study at any time during the trial.

10.5 Recruitment and consent

When a referral letter of a patient suspected of symptomatic gallstones reaches the outpatient department, a research nurse or physician will phone and explain the trial to the patient. Subsequently, study information, the baseline questionnaire and prepaid envelope will be sent to the patient. Thereafter, patients will have the possibility to ask questions. They will be allowed to consider their participation for 2 days. After this period, patients will be contacted and again, they will get the opportunity to ask questions. If the patient wants to participate, (s)he has to send the informed consent form and the baseline questionnaire back to the outpatient department. The patient will be randomized to one of the study arms and subsequently will be seen and treated by a physician.

10.6 Objection by minors or incapacitated subjects

Minors and incapacitated subjects will not be included in this study.

10.7 Benefits and risks assessment, group relatedness

The gallbladder will be removed in both groups if there is a clear medical reason. A burden may be that a patient prefers to be operated immediately, but because we do not know if we help the subject to get rid of his symptoms there will be a possibility that we postpone the eventual operation or drop it if the symptoms have disappeared. Another possible burden of this study is that the completion of the questionnaires may require some extra time of the subject. Patients will receive the same questionnaires at home before treatment and at 3, 6, 9 and 12 months and will be asked to return the completed questionnaires. These questionnaires relate to symptoms and wellbeing of the subjects. This will take about 20 minutes per time.

10.8 Compensation for injury

Since two standard treatments are compared in this study, the Medical Ethical Committee (MEC) has granted dispensation for the obligation to take out an insurance for subjects. The investigator and all participating centers have a liability insurance which is in accordance with article 7, subsection 6 of the WMO.

10.9 Incentives

Subjects will not receive any incentives or reimbursement or travel costs to the hospital.

11. ADMINISTRATIVE ASPECTS, MONITORING AND PUBLICATION

11.1 Handling and storage of data and documents

Design and maintenance of the database for the present study will be performed by the study group. All data will be entered in an electronic CRF. A subject identification code list will be used to link the anonymous data to the subject. The key to the code will be safeguarded by the coordinating investigator. Data will be kept for at least 15 years after the study ending.

11.2 Monitoring and Quality Assurance

11.3 Amendments

Amendments are changes made to the research after a favourable opinion by the accredited METC has been given. All amendments will be notified to the METC that gave a favourable opinion.

All substantial amendments will be notified to the METC and to the competent authority (CA).

Non-substantial amendments will not be notified to the accredited METC and the CA, but will be recorded and filed by the sponsor.

11.4 Annual progress report

The investigator will submit a summary of the progress of the trial to the accredited METC once a year. Information will be provided on the date of inclusion of the first subject, numbers of subjects included and numbers of subjects that have completed the trial, other problems, and amendments.

11.5 End of study report

The investigator will notify the accredited METC of the end of the study within a period of 8 weeks. The end of the study is defined as the last patient's last visit.

In case the study is ended prematurely, the investigator will notify the accredited METC within 15 days, including the reasons for the premature termination.

Within one year after the end of the study, the investigator will submit a final study report

with the results of the study, including any publications/abstracts of the study, to the accredited METC.

11.6 Public disclosure and publication policy

The present study is investigator driven. The investigators will publish the study results in compliance with the prevailing Central Committee on Research Involving Human Subjects (CCMO) publication policy.

12. REFERENCES

1. Everhart JE, Khare M, Hill M, Maurer KR. Prevalence and ethnic differences in gallbladder disease in the United States. *Gastroenterology.* Sep 1999;117(3):632-639.
2. Shaffer EA. Gallstone disease: Epidemiology of gallbladder stone disease. *Best Pract Res Clin Gastroenterol.* 2006;20(6):981-996.
3. Halldestam I, Enell EL, Kullman E, Borch K. Development of symptoms and complications in individuals with asymptomatic gallstones. *Br J Surg.* Jun 2004;91(6):734-738.
4. Heaton KW, Braddon FE, Mountford RA, Hughes AO, Emmett PM. Symptomatic and silent gall stones in the community. *Gut.* Mar 1991;32(3):316-320.
5. www.kiwaprismant.nl.
6. Friedman GD. Natural history of asymptomatic and symptomatic gallstones. *Am J Surg.* Apr 1993;165(4):399-404.
7. Wittenburg H. Hereditary liver disease: gallstones. *Best Pract Res Clin Gastroenterol.* Oct 2010;24(5):747-756.
8. The epidemiology of gallstone disease in Rome, Italy. Part II. Factors associated with the disease. The Rome Group for Epidemiology and Prevention of Cholelithiasis (GREPCO). *Hepatology.* Jul-Aug 1988;8(4):907-913.
9. The epidemiology of gallstone disease in Rome, Italy. Part I. Prevalence data in men. The Rome Group for Epidemiology and Prevention of Cholelithiasis (GREPCO). *Hepatology.* Jul-Aug 1988;8(4):904-906.
10. Berger MY, Olde Hartman TC, van der Velden JJ, Bohnen AM. Is biliary pain exclusively related to gallbladder stones? A controlled prospective study. *Br J Gen Pract.* Aug 2004;54(505):574-579.
11. Berger MY, van der Velden JJ, Lijmer JG, de Kort H, Prins A, Bohnen AM. Abdominal symptoms: do they predict gallstones? A systematic review. *Scand J Gastroenterol.* Jan 2000;35(1):70-76.
12. http://www.plexus.nl/uploads/PDF/nieuwe%20analyse%20rapportage%20indicatoren%20indicatiestelling.pdf.

13. Legorreta AP, Silber JH, Costantino GN, Kobylinski RW, Zatz SL. Increased cholecystectomy rate after the introduction of laparoscopic cholecystectomy. *JAMA.* Sep 22-29 1993;270(12):1429-1432.

14. Lamberts MP, Lugtenberg M, Rovers MM, et al. Persistent and de novo symptoms after cholecystectomy: a systematic review of cholecystectomy effectiveness. *Surg Endosc.* Mar 2013;27(3):709-718.

15. Bates T, Ebbs SR, Harrison M, A'Hern RP. Influence of cholecystectomy on symptoms. *Br J Surg.* Aug 1991;78(8):964-967.

16. Borly L, Anderson IB, Bardram L, et al. Preoperative prediction model of outcome after cholecystectomy for symptomatic gallstones. *Scand J Gastroenterol.* Nov 1999;34(11):1144-1152.

17. Diehl AK, Sugarek NJ, Todd KH. Clinical evaluation for gallstone disease: usefulness of symptoms and signs in diagnosis. *Am J Med.* Jul 1990;89(1):29-33.

18. Gui GP, Cheruvu CV, West N, Sivaniah K, Fiennes AG. Is cholecystectomy effective treatment for symptomatic gallstones? Clinical outcome after long-term follow-up. *Ann R Coll Surg Engl.* Jan 1998;80(1):25-32.

19. Ros E, Zambon D. Postcholecystectomy symptoms. A prospective study of gall stone patients before and two years after surgery. *Gut.* Nov 1987;28(11):1500-1504.

20. Vetrhus M, Berhane T, Soreide O, Sondenaa K. Pain persists in many patients five years after removal of the gallbladder: observations from two randomized controlled trials of symptomatic, noncomplicated gallstone disease and acute cholecystitis. *J Gastrointest Surg.* Jul-Aug 2005;9(6):826-831.

21. Weinert CR, Arnett D, Jacobs D, Jr., Kane RL. Relationship between persistence of abdominal symptoms and successful outcome after cholecystectomy. *Arch Intern Med.* Apr 10 2000;160(7):989-995.

22. de Reuver PR, Sprangers MA, Rauws EA, et al. Impact of bile duct injury after laparoscopic cholecystectomy on quality of life: a longitudinal study after multidisciplinary treatment. *Endoscopy.* Aug 2008;40(8):637-643.

23. Flum DR, Cheadle A, Prela C, Dellinger EP, Chan L. Bile duct injury during cholecystectomy and survival in medicare beneficiaries. *JAMA.* Oct 22 2003;290(16):2168-2173.

24. *NVVH, Evidence-based richtlijn. Onderzoek en behandeling van galstenen. Nederlandse Vereniging voor Heelkunde, 2007.*

25. Nilsson E, Ros A, Rahmqvist M, Backman K, Carlsson P. Cholecystectomy: costs and health-related quality of life: a comparison of two techniques. *Int J Qual Health Care.* Dec 2004;16(6):473-482.

26. Keus F, de Vries J, Gooszen HG, van Laarhoven CJ. Assessing factors influencing return back to work after cholecystectomy: a qualitative research. *BMC Gastroenterol.* 2010;10:12.

27. *Rijksbegroting 2012, artikel 42 Gezondheidszorg, Doelmatigheidsmaatregelen*.

28. Mayumi T, Takada T, Kawarada Y, et al. Results of the Tokyo Consensus Meeting Tokyo Guidelines. *J Hepatobiliary Pancreat Surg.* 2007;14(1):114-121.

29. van Santvoort HC, Besselink MG, de Vries AC, et al. Early endoscopic retrograde cholangiopancreatography in predicted severe acute biliary pancreatitis: a prospective multicenter study. *Ann Surg.* Jul 2009;250(1):68-75.

30. van Ruler O, Mahler CW, Boer KR, et al. Comparison of on-demand vs planned relaparotomy strategy in patients with severe peritonitis: a randomized trial. *JAMA.* Aug 22 2007;298(8):865-872.

31. Lameris W, van Randen A, van Es HW, et al. Imaging strategies for detection of urgent conditions in patients with acute abdominal pain: diagnostic accuracy study. *BMJ.* 2009;338:b2431.

32. de Vries EN, Prins HA, Crolla RM, et al. Effect of a comprehensive surgical safety system on patient outcomes. *N Engl J Med.* Nov 11 2010;363(20):1928-1937.

33. van der Vlies CH, Keulemans YC, Gouma DJ, Boermeester MA. [Patients with upper abdominal pain and echographically-proven gallstones: start with expectative management]. *Ned Tijdschr Geneeskd.* Jul 21 2007;151(29):1605-1609.

34. Mertens MC, De Vries J, Scholtes VP, Jansen P, Roukema JA. Prospective 6 weeks follow-up post-cholecystectomy: the predictive value of pre-operative symptoms. *J Gastrointest Surg.* Feb 2009;13(2):304-311.

35. Mertens MC, Roukema JA, Scholtes VP, De Vries J. Risk assessment in cholelithiasis: is cholecystectomy always to be preferred? *J Gastrointest Surg.* Aug

2010;14(8):1271-1279.

36. Dindo D, Demartines N, Clavien PA. Classification of surgical complications: a new proposal with evaluation in a cohort of 6336 patients and results of a survey. *Ann Surg.* Aug 2004;240(2):205-213.

37. Brooks R. EuroQol: the current state of play. *Health Policy.* Jul 1996;37(1):53-72.

38. Eypasch E, Williams JI, Wood-Dauphinee S, et al. Gastrointestinal Quality of Life Index: development, validation and application of a new instrument. *Br J Surg.* Feb 1995;82(2):216-222.

39. van der Kloot WA, Oostendorp RA, van der Meij J, van den Heuvel J. [The Dutch version of the McGill pain questionnaire: a reliable pain questionnaire]. *Ned Tijdschr Geneeskd.* Apr 1 1995;139(13):669-673.

40. Bloechle C, Izbicki JR, Knoefel WT, Kuechler T, Broelsch CE. Quality of life in chronic pancreatitis: results after duodenum-perserving resection of the head of the pancreas. Pancreas 1995:11; 77-85.

41. http://www.bmg.eur.nl/fileadmin/ASSETS/bmg/english/iMTA/Publications/Manuals Questionnaires/Handleiding._SF-HLQ_september_2010_definitief.pdf. http://eprints.gla.ac.uk/4148/1/Briggs4148.pdf.

42. Mauskopf JA, Sullivan SD, Annemans L, et al. Principles of good practice for budget impact analysis: report of the ISPOR Task Force on good research practices--budget impact analysis. *Value Health.* Sep-Oct 2007;10(5):336-347.

43. *http://www.rijksbegroting.nl/2012/voorbereiding/begroting,kst160371_8, sections 42.2.2. and 42.2.3*.

44. *Westert GP. [Less care, more quality: towards less unnecessary care and reduced inappropriate practice variation in hospital care provision]. Nijmegen: UMC St. Radboudl/IQScientific Institute for Quality of Health Care, June 2012*.

45. Schmidt et al. Post-cholecystectomy symptoms were caused by persistence of a functional gastrointestinal disorder. World J Gastroenterol. 2012. 18(12): 1365–1372.
