# Supplementary material for: Restrictive Strategy vs Usual Care for Cholecystectomy in Patients With Abdominal Pain and Gallstones: 5-Year Follow-Up of the SECURE Randomized Clinical Trial
Source: JAMA Surg. 2024 Aug 21;159(11):1235–43. doi: 10.1001/jamasurg.2024.3080 (PMC11339699; doi:10.1001/jamasurg.2024.3080)
Supplement: Supplement 2. — Protocol amendment 1 [file jamasurg-e243080-s002.pdf]

Amendement 1. Scrutinizing (in)efficient use of cholecystectomy: a randomized trial concerning variation in practice (SECURE-trial)

Long term follow-up

Five years after inclusion, patients will be approached to fill-out a long term follow-up questionnaire. Only patients who gave permission on the informed consent form to be contacted again (after initial 1 year follow-up of the trial), will be send the questionnaire. By the follow-up after five years, consistency of the effect of the restrictive strategy for gallbladder removal at long term can be evaluated. The primary and secondary endpoints as described for the 12 month follow-up of the trial, will be established at five year follow-up. Additional secondary outcomes are the healthcare consumption for gallstone symptoms and persistent abdominal symptoms after cholecystectomy.

The long term questionnaire will consist of the same questionnaires as the 12 month follow-up questionnaire (Izbicki Pain Score, The Gallstone Symptom List, EuroQol 5 Dimensions (EQ-5D), the Short-Form Health and Labour Questionnaire (SF-HLQ), Gastrointestinal Quality of Life Index (GIQLI)) and an additional validated questionnaire on healthcare consumption for gallstone symptoms or persistent abdominal pain after cholecystectomy. This is the Patients' Experience of Surgery Questionnaire (PESQ). The PESQ consists of general questions on results of surgery, abdominal symptoms and related healthcare use. [1]

1. Black, N. and C. Sanderson, *Day surgery; development of a questionnaire for eliciting patients' experiences.* Qual Health Care, 1993. **2**(3): p. 157-61.
