# Supplementary material for: Restrictive Strategy vs Usual Care for Cholecystectomy in Patients With Abdominal Pain and Gallstones: 5-Year Follow-Up of the SECURE Randomized Clinical Trial
Source: JAMA Surg. 2024 Aug 21;159(11):1235–43. doi: 10.1001/jamasurg.2024.3080 (PMC11339699; doi:10.1001/jamasurg.2024.3080)
Supplement: Supplement 3. — Protocol amendment 2 [file jamasurg-e243080-s003.pdf]

Amendement 2. Scrutinizing (in)efficient use of cholecystectomy: a randomized trial concerning variation in practice (SECURE-trial)

Additional Secondary outcomes

Patient-reported satisfaction on treatment outcome

At 12 month follow-up, all patients will be asked to express their satisfaction with the treatment outcome (either surgical or conservative) with a Numerical Rating Scale (NRS) between 0 and 10 (0 being the worst and 10 being the best appraisal). The satisfaction on the treatment will be reported, as mean with standard deviation if the data are normally distribution, and as median with interquartile range if the data are skewed. The independent T-test or Mann-Whitney test will be used to compare the outcome between study arms.

Alternative diagnostics and treatment

At 12 month follow-up, information on diagnostic procedures and treatments for abdominal pain will be obtained for all patients, by patient's interview and patient's medical records. For the conservatively treated patients (i.e. who did not undergo cholecystectomy), the proportion of patients that underwent alternative diagnostic procedures and/or treatments will be reported. The proportion of patients for which an alternative diagnosis for the abdominal pain was found, and type of diagnoses, will be reported.
