# Supplementary material for: Restrictive Strategy vs Usual Care for Cholecystectomy in Patients With Abdominal Pain and Gallstones: 5-Year Follow-Up of the SECURE Randomized Clinical Trial
Source: JAMA Surg. 2024 Aug 21;159(11):1235–43. doi: 10.1001/jamasurg.2024.3080 (PMC11339699; doi:10.1001/jamasurg.2024.3080)
Supplement: Supplement 4. — eMethods. Non-inferiority testing in per protocol analysis eResults. Patients with and without cholecystectomy eTable. Comparison of patients with and without cholecystectomy after 5-year follow-up [file jamasurg-e243080-s004.pdf]

Supplemental Online Content

Comes DJ, Wennmacker SZ, Latenstein CSS, et al. Restrictive strategy vs usual care for cholecystectomy in patients with abdominal pain and gallstones: 5-year follow-up of the SECURE randomized clinical trial. *JAMA Surg*. Published online August 21, 2024. doi:10.1001/jamasurg.2024.3080

eMethods. Non-inferiority testing in per protocol analysis
eResults. Patients with and without cholecystectomy
eTable. Comparison of patients with and without cholecystectomy after 5-year follow-up

This supplemental material has been provided by the authors to give readers additional information about their work.

© 2024 Comes DJ et al. *JAMA Surgery.*

eMethods
Non-inferiority testing in per protocol analysis

In the per-protocol analysis, non-inferiority at five-year follow-up was again not shown , 61.7% of patients were pain-free in the usual care group, compared to 62.6% of patients in the restrictive strategy group (difference, -1.0%; one-sided 95% lower confidence limit, -7.6%; p-non-inferiority=0.157).

The non-inferiority of being pain-free, defined as a VAS score of ≤ 4, was tested in the per-protocol analysis group. At five-year follow-up, 81.3% of patients were pain-free in the usual care group, compared to 80.8% of patients in the restrictive strategy group (difference, 0.6%; one-sided 95% lower confidence limit, -4.0%; p-non-inferiority=0.022).

eResults
Patients with and without cholecystectomy

In Table 4 (Appendix) patients with and without cholecystectomy were compared. In the cholecystectomy group, surgery was performed after a median period of 6.0 weeks after randomization (IQR 3.0-13.0), the conversion rate to open surgery was 1.8% (15/824) and in 1.3% (11/824) a bile duct injury occurred. In total, 62 patients underwent elective cholecystectomy during the 1 to 5-year follow-up period, with the following indications: recurrent biliary colic (40/62), acute cholecystitis (13/62), choledocholithiasis (8/62), biliary pancreatitis (1/62), and 1 patient with acute cholecystitis who underwent cholecystectomy in emergency setting.

After five-year follow-up a total of 347/588 patients (59.0%) in cholecystectomy group were pain-free compared to 96/174 patients (55.2%) in the conservative treatment group (p=0.367). A VAS pain score ≤ 4 was reported by 80.7% of patients after cholecystectomy versus 77.9% of patients with conservative treatment (P=0.379). No differences were observed in terms of biliary symptoms between both groups, but in patients with cholecystectomy significantly more intolerance of fatty foods and diarrhea was reported (10.2% vs. 4.6%, p=0.015, and 9.5 vs. 4.6%, p=0.027, respectively).

© 2024 Comes DJ et al. *JAMA Surgery.*

eTable. Comparison of patients with and without cholecystectomy after 5-year follow-up

	5 year		
	Cholecystectomy (n=824)	Conservative treatment (n=241)	P
**Patient-reported outcomes**			
Pain-free[A] – (%)[B]	347 (63.3)	96 (57.5)	0.174
VAS ≤4[C] – (%)[D]	609 (80.7)	166 (77.9)	0.379
**Biliary symptoms**			
Severe pain in attacks (%)^&	133 (16.1)	37 (15.4)	0.426
Located in the right upper quadrant and/or epigastrio (%)^&	115 (13.9)	37 (15.4)	0.501
Pain radiating to the back (%)^	73 (8.9)	23 (9.5)	0.688
Pain responding to simple analgesics (%)^	28 (3.4)	11 (4.6)	0.976
Duration of pain longer than 15-30 min (%)^&	120 (14.6)	34 (14.1)	0.683
Biliary colic& (%)	77 (9.3)	23 (9.5)	0.957
Fulfilment of all 5 (%)	19 (2.3)	7 (2.9)	0.672
**Functional symptoms**			
Intolerance of fat foods (%)	84 (10.2)	11 (4.6)	0.015
Nausea and vomiting (%)	36 (4.4)	7 (2.9)	0.386
Diarrhea (%)	78 (9.5)	11 (4.6)	0.027
Difficult defecation (%)	45 (5.5)	13 (5.4)	0.858
Acid burn (%)	75 (9.1)	15 (6.2)	0.238
Abdominal bloating (%)	77 (9.3)	24 (10.0)	0.551
**Patient-reported satisfaction median [IQR]**	9.1 (7.0-10.0)	8.0 (5.0-9.7)	<.001
Additional treatment and diagnosis			
Time to cholecystectomy, median [IQR], weeks	6.0 (3.0-13.0)	NA	
**Total visits of outpatient clinic due to persisted**	129 (15.8)	37 (15.4)	0.876
**abdominal** symptoms	71 (8.7)	18 (7.5)	0.552
Department of Surgery (%)	82 (10.0)	23 (9.5)	0.822
Department of Gastroenterology (%)			
Imaging (US and/or CT) (%)	146 (17.9)	29 (12.1)	0.036
Upper Gastrointestinal Endoscopy (%)	38 (4.7)	11 (4.6)	0.949
Diagnosed Functional Gastrointestinal disorders, yes (%)	56 (6.9)	16 (6.7)	0.924

Abbreviations US=Ultrasound; CT=Conventional Tomography; FGID=Functional Gastrointestinal Disorders.
Data on secondary outcomes from patient-reported outcomes were available for 491 patients in the usual care group and 479 patients in the restrictive strategy group.
[A] Pain-free was defined as Izbicki pain score ≤10 and VAS ≤4
[B] Assessed in 716 patients
[C] VAS score of 4 points or lower
[D] Assessed in 968 patients, because data of 2 patients on treatment was missing.
[E] Total number of patients who either visit the outpatient clinic of Surgery or Gastroenterology or both

© 2024 Comes DJ et al. *JAMA Surgery.*
