# Supplementary material for: Restrictive Strategy vs Usual Care for Cholecystectomy in Patients With Abdominal Pain and Gallstones: 5-Year Follow-Up of the SECURE Randomized Clinical Trial
Source: JAMA Surg. 2024 Aug 21;159(11):1235–43. doi: 10.1001/jamasurg.2024.3080 (PMC11339699; doi:10.1001/jamasurg.2024.3080)
Supplement: Supplement 5. — Data sharing statement [file jamasurg-e243080-s005.pdf]

Data Sharing Statement

Comes. Restrictive Strategy vs Usual Care for Cholecystectomy in Patients With Abdominal Pain and Gallstones. *JAMA Surg*. Published August 21, 2024.
doi:10.1001/jamasurg.2024.3080

Data
Data available: Yes
Data types: Deidentified participant data
How to access data: provide complete email address if request for data must be sent to an individual.
When available: With publication

Supporting Documents
Document types: Statistical/analytic code
How to access documents: https://pubmed.ncbi.nlm.nih.gov/30390706/
https://pubmed.ncbi.nlm.nih.gov/27411788/
When available: With publication

Additional Information
Who can access the data: researchers whose proposed use of the data has been approved
Types of analyses: Only for specified purpose.
Mechanisms of data availability: with investigator support,after approval of a proposal, and with a signed data access agreement
